# Supplementary material for: Evidence from Finland and Sweden on the relationship between early-life diseases and lifetime childlessness in men and women
Source: Nat Hum Behav. 2023 Dec 18;8(2):276–87. doi: 10.1038/s41562-023-01763-x (PMC10896715; doi:10.1038/s41562-023-01763-x)
Supplement: Supplementary file 1 — Supplementary Figs. 1–17, Tables 1–6, 8 and 10, and Notes. [file 41562_2023_1763_MOESM1_ESM.pdf]

nature human behaviour

Article

https://doi.org/10.1038/s41562-023-01763-x

Evidence from Finland and Sweden on the relationship between early-life diseases and lifetime childlessness in men and women

In the format provided by the authors and unedited

Table of Contents

List of Supplementary Figures……………..……..…....………………………………………………1

List of Supplementary Tables.………..…………..………………..……..……..…....……………………..3

1. Theoretical framework for the relationship of diseases with childlessness..………….……..……...…4
2. Review of the literature linking diseases to childlessness.………..……………..……..………..……...…6
3. Descriptive statistics of fertility and social characteristics in Finland and Sweden…………...……...9
   3.1. Background of childlessness and feritlity in Finland and Sweden………...……………….....…..9
   3.2. Descriptive statistics of childlessness and feritlity in Finland and Sweden ………......…...…...9
   3.3. Descriptive statistics of social characteristics in Finland and Sweden…………..……….......10
4. Disease diagnosis….………………….……………………….……..………………………………..13
   4.1. Definition of disease endpoints through ICD-8, ICD-9, and ICD-10……..….……….....……..13
   4.2. Diseases not included due to virtually complete lack of children……………......…………..…..36
   4.3. Disease prevalence from Finland and Sweden………………….....…….……………….……..37
5. Relationship of disease diagnoses with childlessness..……………....……...……………….......…38
   5.1. Effects of each disease diagnosis……………..........………………..………….……..….......…38
   5.2. Effects by disease category……………....………………...……………....…………………...39
   5.3. Age-of-onset-dependent effects……………...………………...…………………….…...……40
   5.4 Stratification by parity for individuals with children...………………..……....…...………....41
6. Mediation effect by singlehood………...……….......……....………………….....……….……….....43
   6.1. Effects on singlehood…………………………..……..………………....…………….…...…44
   6.2. Mediation analysis……………..…….……….....………......……..……….....………...….....…46
   6.3. Effects among individuals with registered partner……….………..…………………..……..…47
7. Population-based and sensitivity analyses……………….....……………....………….....….............49

Reference…………………………………………………....……………...…………......……..58

List of Supplementary Figures

Supplementary **Fig. 1**: Theoretical framework for the relationship of diseases with childlessness.

Supplementary **Fig. 2**: Number of disease endpoints by disease category.

Supplementary **Fig. 3**: Prevalence by age 45 in women (panel **a**) and 50 in men (panel **b**) for 213 disease diagnoses that were available in both Finland and Sweden. Each dot represents a disease diagnosis. Colors are assigned by disease categories.

Supplementary **Fig. 4**: Relationship of disease diagnoses with childlessness by age 45 in women (panel **a**, **c**, and **e**) and 50 in men (panel **b**, **d**, and **f**) from main analysis (childless individuals were compared to those with at least one child) versus the analysis stratified by parities (childless individuals were compared to those with one child (panel **a** and **b**), with two children (panel **c** and **d**), or with at least three or more children (panel **e** and **f**)), for disease diagnoses that were significantly increased childlessness from the main analysis.

Supplementary **Fig. 5**: Relationship of 402 disease diagnoses with being without a partner by age 45 in women (panel **a**) and 50 in men (panel **b**). Only disease diagnoses that are significantly associated after multiple-testing correction are colored.

Supplementary **Fig. 6**: Sex-specific effects for the association between disease diagnoses and being without a partner by age 45 in women and 50 in men, for 68 disease diagnoses that were significantly associated with increased childlessness from the main analysis. Only disease diagnoses that are significantly different between sexes at a nominal P value (no adjustments were made for multiple comparisons) with a two-sided t-test are colored.

Supplementary **Fig. 7**: Relationship of disease diagnoses with childlessness (main analysis) versus singlehood by age 45 in women (panel **a**) and 50 in men (panel **b**), for 74 disease diagnoses that were significantly associated with childlessness from the main analysis. Only disease diagnoses that are significantly different between two outcomes at a nominal P value (no adjustments were made for multiple comparisons) with a two-sided t-test are colored.

Supplementary **Fig. 8**: Relationship of 169 disease diagnoses with childlessness among women (by age 45) (panel **a**) and men (by age 50) (panel **b**) with registered partners. Only disease diagnoses that are significantly associated after multiple-testing correction are colored.

Supplementary **Fig. 9**: Relationship of disease diagnoses with childlessness by age 45 in women (panel **a**) and 50 in men (panel **b**) from the general population (main analysis) versus people with registered partners, for 36 disease diagnoses that were significantly associated with childlessness from the main analysis.

Supplementary **Fig. 10**: Relationship of 403 disease diagnoses with childlessness by age 45 in women (panel **a**) and 50 in men (panel **b**) from using a nested incident-matched case-control design, matched by birth year, birth municipality, and highest parental education level. Only disease diagnoses that are significantly associated after multiple-testing correction are colored.

Supplementary **Fig. 11**: Relationship of disease diagnoses with childlessness by age 45 in women (panel **a**) and 50 in men (panel **b**) from the sibling-based (main analysis) versus population-based analyses, for 74 disease diagnoses that were significantly associated with childlessness from the main analysis.

Supplementary **Fig. 12**: Relationship of 365 disease diagnoses with childlessness restricted to women alive by age 45 (panel **a**) and men by 50 (panel **b**), using a sibling-based design. Only disease diagnoses that are significantly associated after multiple-testing correction are colored.

Supplementary **Fig. 13**: Relationship of disease diagnoses with childlessness in the general population (main analysis) versus the sensitivity analysis which restricted to women alive by age 45 (panel **a**) and men by 50 (panel **b**), for 71 disease diagnoses that were significantly associated with childlessness from the main analysis.

Supplementary **Fig. 14**: Relationship of 403 disease diagnoses with childlessness by age 45 in women (panel **a**) and 50 in men (panel **b**) from using a Cox proportional hazards regression model stratified

by same-sex full-siblings. Only disease diagnoses that are significantly associated after multiple-testing correction are colored.

Supplementary **Fig. 15**: Relationship of disease diagnoses with childlessness by age 45 in women (panel **a**) and 50 in men (panel **b**) from conditional logistic regression (main analysis) versus stratified Cox proportional hazards regression, for 74 disease diagnoses that were significantly associated with childlessness from the main analysis.

Supplementary **Fig. 16**: Relationship of disease diagnoses with childlessness by age 45 in women (panel **a**) and 50 in men (panel **b**) from main analysis versus sensitivity analysis adjusting for individuals' highest education level, for 74 disease diagnoses that were significantly associated with childlessness from the main analysis.

Supplementary **Fig. 17**: Relationship of disease diagnoses with childlessness by age 45 in women (panel **a**) and 50 in men (panel **b**) from population-based analyses with or without adjusting for individuals' highest education level, for 74 disease diagnoses that were significantly associated with childlessness from the main analysis.

List of Supplementary Tables

Supplementary **Table 1**: Summary of previous studies linking the relationship of diseases with childlessness.

Supplementary **Table 2**: Descriptive statistics of childlessness and parity for index persons in Finland and Sweden.

Supplementary **Table 3**: Descriptive statistics of social characteristics for index persons in Finland.

Supplementary **Table 4**: Descriptive statistics of social characteristics for index persons in Sweden.

Supplementary **Table 5**: Definition of 414 disease endpoints (16 categories) through ICD-8, ICD-9, and ICD-10.

Supplementary **Table 6**: Diseases not included in the analysis due to resulting in an almost complete lack of children.

Supplementary **Table 7**: Relationship of disease diagnoses with childlessness by age 45 in women and 50 in men, with a two-sided test.

Supplementary **Table 8**: Relationship of disease diagnoses with childlessness by age 45 in women and 50 in men by disease category, with a two-sided test.

Supplementary **Table 9**: Age-of-onset-dependent effects for each disease diagnosis that significantly increased childlessness, with a two-sided test.

Supplementary **Table 10**: Age-of-onset-dependent effects across all disease diagnoses that significantly increased childlessness, with a two-sided test.

Supplementary **Table 11**. Relationship of disease diagnoses with childlessness by age 45 in women and 50 in men, stratified by parity of individuals with children, with a two-sided test.

Supplementary **Table 12**: Direct and indirect effects (mediated by singlehood) of disease on childlessness estimated from mediation analysis, with a two-sided test.

1. Theoretical framework for the relationship of diseases with childlessness

In this study childlessness is used to describe individuals that have had no live-born children by the end of their entire reproductive lifespan (age 45 for women; 50 for men). Childlessness is defined in the literature as being both involuntary (e.g., infertility, inability to find a partner) and voluntary or 'childfree'[1](e.g., active choice, preference[2]) (see Text Box Main article).

Diseases can influence individuals' chances of being childless over their lifetime (Supplementary **Fig. 1**). First, diseases may directly influence childlessness through medical conditions that affect the fecundity and mortality of the individual as well as the risk of stillbirth[3]. Second, diseases can impact selection into a partnership which in turn lowers the chance of being partnered and thus having children[4]. Also, some may choose to be childfree due to the concern of genetic transmission of certain known genetic conditions[5]. There could also be concerns about intergenerational transmission of disease and the intergenerational transmission of health and fertility preferences. Finally, diseases are associated with lower socio-economic status, unemployment, and economic uncertainty, which may amplify the psychosocial impact of diseases on childlessness, possibly by increasing partnership instability or reducing fertility intentions[6]. There are of course multiple non-disease related reasons that individuals postpone or not having children related to factors such as individual choice, work-life reconciliation, housing and economic uncertainty, or inability to find a partner. While individuals' health conditions can also be influenced by their childless status[7,8], our study focused on the path from early-life diseases to lifetime chances of being childless.

Supplementary **Fig. 1**: Theoretical framework for the relationship of diseases with childlessness.

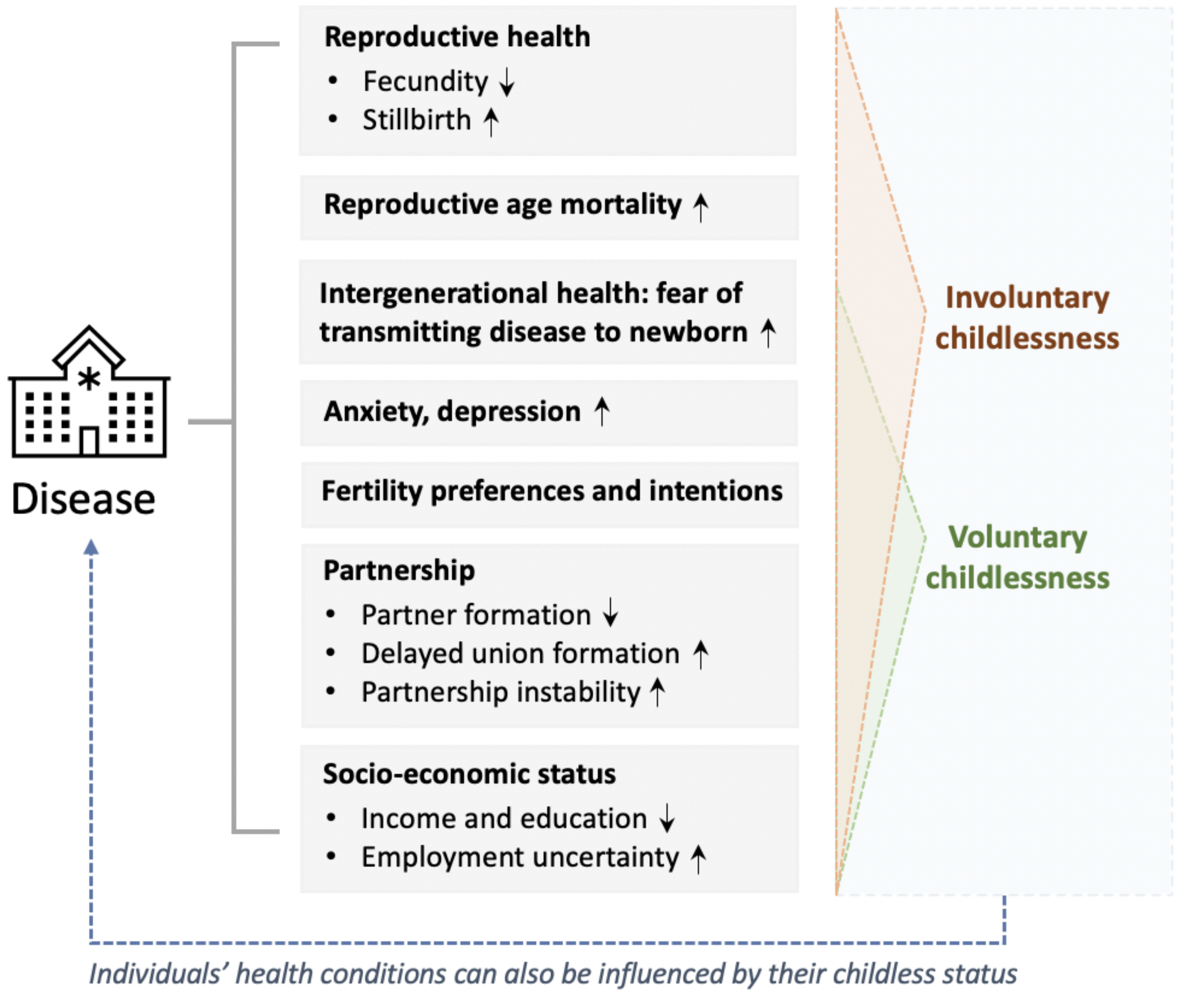

2. Review of the literature linking diseases to childlessness

Supplementary **Table 1**: Summary of previous studies linking the relationship of diseases with childlessness.

Study	Country	Data	Disease	Outcome	Review
Fear and Fertility in Inflammatory Bowel Disease: A Mismatch of Perception and Reality Affects Family Planning Decisions[9]	Australia	IBD-database (hospital-based)	Crohn's disease, IBD	Childlessness	No
Voluntary childlessness is increased in women with inflammatory bowel disease[10]	US	Survey among members of Crohn's and Colitis Foundation of America	Crohn's disease, IBD	Voluntary childlessness, temporary childlessness	No
Tubal factor infertility, with special regard to chlamydial salpingitis[11]	--	--	Tubal factor infertility	Involuntary childlessness	Yes
Systematic review: fertility in non-surgically treated inflammatory bowel disease[12]	--	--	Crohn's disease, IBD	Fertility, childlessness	Yes
What Factors Might Drive Voluntary Childlessness (VC) in Women with IBD? Does IBD-specific Pregnancy-related Knowledge Matter?[13]	UK	Survey among members of Crohn's and Colitits UK	Crohn's disease, IBD	Voluntary childlessness	No
Idiopathic impaired spermatogenesis: genetic epidemiology is unlikely to provide a short-cut to better understanding[14]	--	--	Impaired spermatogenesis	--	No
Does the level of reproductive knowledge specific to inflammatory bowel disease predict childlessness among women with inflammatory bowel disease?[15]	Canada	Crohn’s and Colitis pregnancy knowledge questionnaire	Crohn's disease, IBD	Childlessness	No
Sexual and reproductive issues and inflammatory bowel disease: a neglected topic in men[16]	--	--	Crohn's disease, IBD	Childlessness	Yes
Pregnancy outcomes in women with inflammatory bowel disease: A 10-year nationwide population-based cohort study[17]	South Korea	Korean national health insurance claims database	Crohn's disease, IBD	Pregnancy outcomes	No
Definitive childlessness in women with multiple sclerosis: a multicenter study[18]	Italy	Hospital patients	Multiple sclerosis	Childlessness	No
Sexual health and fertility for individuals with inflammatory bowel disease[19]	--	--	Crohn's disease, IBD	Childlessness	Yes
Inflammatory bowel disease-specific pregnancy knowledge of gastroenterologists against general practitioners and obstetricians[20]	Australia	Crohn’s and Colitis Pregnancy Knowledge questionnaire	Crohn's disease / IBD	Childlessness	No
Systematic review: the consequences of psychosocial effects of inflammatory bowel disease on patients’ reproductive health[21]	--	--	Crohn's disease / IBD	Childlessness	Yes

Endometriosis[22]	--	--	Endometriosis	Infertility	Yes
ESHRE guideline: management of women with endometriosis[23]	--	--	Endometriosis	Infertility	Yes
Polycystic ovary syndrome[24]	--	--	Polycystic ovary syndrome	Infertility	Yes
Diagnosis and Treatment of Polycystic Ovary Syndrome: An Endocrine Society Clinical Practice Guideline[25]	--	--	Polycystic ovary syndrome	Infertility	Yes
Endometriosis: pathogenesis and treatment[26]	--	--	Endometriosis	Infertility	Yes
Polycystic ovary syndrome: etiology, pathogenesis and diagnosis[27]	--	--	Polycystic ovary syndrome	Infertility	Yes
Coeliac disease[28]	--	--	Coeliac disease	Infertility	Yes
Family planning in inflammatory bowel disease: childlessness and disease-related concerns among female patients[29]	Germany, Austria, Switzerland	University-hospital based survey	Crohn's disease / IBD	Childlessness	No
Risk Factors for Voluntary Childlessness in Men and Women With Inflammatory Bowel Disease[30]	US	Survey among patients	Crohn's disease / IBD	Voluntary childlessness	No
Fecundity, pregnancy outcomes, and breastfeeding in patients with inflammatory bowel disease: a large cohort survey[31]	Spain	Survey among patients	Crohn's disease / IBD	Fertility, pregnancy outcomes, breastfeeding	No
Increased risk of preterm birth for women with inflammatory bowel disease[32]	US, North Carolina	Self-administered questionnaire	Crohn's disease / IBD, ulcerative colitis	Voluntary and involuntary childlessness, infertility, fecund-ability, methods of birth control	No
Perception of Reproductive Health in Women with Inflammatory Bowel Disease[33]	Malta, Greece, Israel, Italy, Portugal, and Spain	Survey in hospitals	Crohn's disease / IBD, ulcerative colitis	--	No
Fertility and Assisted Reproductive Technologies Outcomes of Women with Non-surgically Managed Inflammatory Bowel Diseases: A Systematic Review[34]	--	--	Crohn's disease / IBD, ulcerative colitis	Voluntary childlessness, number of children	Yes
The Young Adult with Diabetes: Impact of the Disease on Marriage and Having Children[35]	US	Small survey (N=50)	Diabetis	Marriage, having children	No
The Relationship Between Common Variant Schizophrenia Liability and Number of Offspring in the UK Biobank[36]	UK	Biobank	Schizophrenia	Childlessness	No

Schizophrenia risk and reproductive success: a Mendelian randomization study[37]	UK	Biobank	Schizophrenia liability	Childlessness	No
Early-onset major depressive disorder in men is associated with childlessness[38]	US	National epidemiologic survey on alcohol and related conditions	Major depressive disorder, bipolar disorder	Childlessness, number of children	No

3. Descriptive statistics of fertility and social characteristics in Finland and Sweden

3.1 Background of childlessness and feritlity in Finland and Sweden

Childlessness is interesting to study in the Finnish and Swedish context. Despite declines in period fertility since around 2010, cohort fertility in Finland and Sweden has remained relatively stable and close to replacement levels[39-41]. They have had higher levels of gender equity, rates of female labour force participation, and experienced a strong shift in women's educational expansion[42]. Although fertility has remained relatively high, Finland has had the highest levels of childlessness in Europe[43] and a 'parity polarisation' of high levels of childlessness coupled with higher parities in certain groups[42,44]. There is also a parallel 'partner polarisation', or bifurcation of those who never partner with an increase in multi-partner fertility, given that a new union encourages childbearing[41,45].

Finland differs from Sweden in that it has a parity polarisation amongst particularly the lower, but also the medium-educated groups[42]. These differences have been attributed to Finland's skewed national and local sex ratios related to deaths during WWII, mass emigration, low population density, and a larger proportion of women compared to men with tertiary education[43]. The 'two-child family norm' only seems to hold for the higher educated, and childlessness is higher amongst lower educated men and higher educated women[42]. Nordic gender equality and work-family reconciliation therefore seems to disproportionately aid the highly educated groups to reach the two-child norm. Conversely, the fertility patterns of lower educated men and women in Sweden and Finland are more heterogeneous and polarized between childlessness and higher parity[42].

3.2 Descriptive statistics of childlessness and feritlity in Finland and Sweden

For index individuals in both Finland and Sweden, we considered the numbers of biological children they had by age 45 in women and 50 in men (Supplementary **Table 2**). Among 1,425,640 index women and 1,119,380 men, 230,198 women and 279,454 men were childless. The proportion of childless individuals – those with no live-born children – was higher in men (25.4%) than women (16.6%), and higher amongst Finns (23.3%) than Swedes (18.7%). Among index persons with children, a two-child parity was most prevalent in men (47.1%) and women (48.9%), amongst both Finns (44.0%) and Swedes (50.8%).

Supplementary **Table 2**: Descriptive statistics of childlessness and parity for index persons in Finland and Sweden.

	**Finland**		**Sweden**	
	**Women**	**Men**	**Women**	**Men**
Childlessness	107,913 (18.8 %)	128,213 (27.7 %)	122,285 (14.3%)	151,241 (23.1 %)
One child	91,114 (15.9 %)	69,047 (14.9 %)	115,262 (13.5%)	91,426 (13.9 %)
Two children	206,436 (36.1 %)	145,292 (31.4 %)	377,564 (44.3%)	250,122 (38.1 %)
Three children	114,966 (20.1 %)	81,764 (17.6 %)	177,225 (20.8%)	118,518 (18.1 %)
At least four children	52,089 (9.1 %)	39,094 (8.4 %)	60,786 (7.1%)	44,663 (6.8 %)
Total	572,518 (100%)	463,410 (100%)	853,122 (100%)	655,970 (100%)

3.3 Descriptive statistics of social characteristics in Finland and Sweden

To understand the extent to which the social characteristics varied amongst childless individuals and individuals with children in the general population, we considered the highest education level and parental education level in both Finns and Swedes and partnership patterns in Finns only (Supplementary **Table 3-4**), depending on data availability.

Overall, in both countries, women were more educated than men (e.g., in Finland, 49.8% of index women completed the first stage of tertiary education while the proportion dropped to 32.5% for index men). Both women and men with the lowest educational attainment (level 0-2; pre-primary education, primary education or first stage of basic education, or lower secondary or second stage of basic education) were more likely to be childless (e.g., in Finland, 24.2% were childless in women and 37.4% in men) than the general population (e.g., in Finland, 18.8% were childless in women and 27.7% in men). Amongst individuals who completed the first stage of tertiary education (levels 5 and 6), men were less likely to be childless (e.g., in Finland, 20.8%) compared to the general population (e.g., in Finland, 27.7%), however, for women, a slightly higher probability of childlessness was observed (e.g., in Finland, 19.2%, compared to 18.8% in the general population).

Across generations, individuals included in these analyses were more educated than their parents (e.g., in Finland, 42.1% of index persons completed the first stage of tertiary education while the proportion dropped to 20.5% in their parents). The education level of parents was also correlated with an individual's childless status. For example, men who had parents with the lowest educational attainment (level 0-2) were more likely to be childless (e.g., in Finland, 28.9%) than the general population (e.g., in Finland, 27.7%). Among index persons whose parents had completed the first stage of tertiary education (levels 5-6), men were less likely to be childless (e.g., in Finland, 25.3%) compared to the general population (e.g., in Finland, 27.7%) while women behaved in the opposite way (e.g., in Finland, 21.3%, compared to 18.8% in the general population).

Regarding the partnership, we only obtained detailed information on partnership types for Finns, for which most of the registered relationships were marriages.

Supplementary **Table 3**: Descriptive statistics of social characteristics for index persons in Finland.

Variable	Women		Men	
	**With children**	**Childless**	**With children**	**Childless**
**Parental education level (ISCED 1997)**				
Pre-primary education, primary education or first stage of basic education, or lower secondary or second stage of basic education (Level 0-2)	224,817 (48.4%)	48,904 (45.3%)	177,274 (52.9%)	72,107 (56.2%)
Upper secondary education (Level 3)	141,466 (30.4%)	32,438 (30.1%)	92,034 (27.5%)	33,818 (26.4%)
Post-secondary non-tertiary education (Level 4)	399 (0.1%)	90 (0.1%)	95 (0%)	24 (0%)
First stage of tertiary education (Level 5)	95,139 (20.5%)	25,710 (23.8%)	64,076 (19.1%)	21,735 (17.0%)
Second stage of tertiary education (Level 6)	2,784 (0.6%)	771 (0.7%)	1,718 (0.5%)	529 (0.4%)
**Index person's education level (ISCED 1997)**				
Pre-primary education, primary education or first stage of basic education, or lower secondary or second stage of basic education (Level 0-2)	41,466 (8.9%)	13,232 (12.3%)	52,867 (15.8%)	31,625 (24.7%)
Upper secondary education (Level 3)	184,774 (39.8%)	38,776 (35.9%)	157,338 (46.9%)	64,247 (50.1%)
Post-secondary non-tertiary education (Level 4)	7,992 (1.7%)	1,170 (1.1%)	5,800 (1.7%)	965 (0.8%)
First stage of tertiary education (Level 5)	224,983 (48.4%)	53,117 (49.2%)	115,052 (34.3%)	30,419 (23.7%)
Second stage of tertiary education (Level 6)	5,390 (1.2%)	1,618 (1.5%)	4,140 (1.2%)	957 (0.7%)
**Partnership pattern**				
No partner	69,263 (14.9%)	70,300 (65.1%)	45,308 (13.5%)	93,067 (72.6%)
Ever-married	395,127 (85.0%)	37,169 (34.4)	289,834 (86.5%)	34,719 (27.1%)
Registered partnership	215 (0.05%)	444 (0.41%)	55 (0.02%)	427 (0.33%)

Supplementary **Table 4**: Descriptive statistics of social characteristics for index persons in Sweden.

Variable	Women		Men	
	**With children**	**Childless**	**With children**	**Childless**
**Parental education level (ISCED 1997)**				
Pre-primary education, primary education or first stage of basic education, or lower secondary or second stage of basic education (Level 0-2)	227,829 (31.2%)	38,260 (31.3%)	179,183 (35.5%)	58,593 (38.7%)
Upper secondary education (Level 3)	331,431 (45.3%)	52,960 (43.3%)	217,967 (43.2%)	62,713 (41.5%)
Post-secondary non-tertiary education (Level 4)	13,745 (1.9%)	2,460 (2.0%)	7,705 (1.5%)	2,131 (1.4%)
First stage of tertiary education (Level 5)	149,146 (20.4%)	26,934 (22.0%)	94,453 (18.7%)	26,311 (17.4%)
Second stage of tertiary education (Level 6)	8,686 (1.2%)	1,671 (1.4%)	5,421 (1.1%)	1,493 (1.0%)
**Index person's education level (ISCED 1997)**				
Pre-primary education, primary education or first stage of basic education, or lower secondary or second stage of basic education (Level 0-2)	53,253 (7.3%)	14,833 (12.1%)	71,808 (14.2%)	31,105 (20.6%)
Upper secondary education (Level 3)	362,598 (49.6%)	55,670 (45.5%)	274,402 (54.4%)	80,326 (53.1%)
Post-secondary non-tertiary education (Level 4)	39,626 (5.4%)	7,938 (6.5%)	49,607 (9.8%)	13,314 (8.8%)
First stage of tertiary education (Level 5)	267,718 (36.6%)	42,304 (34.6%)	101,521 (20.1%)	24,897 (16.5%)
Second stage of tertiary education (Level 6)	7,642 (1.0%)	1,540 (1.3%)	7,391 (1.5%)	1,599 (1.1%)

4. Disease diagnosis

4.1. Definition of disease endpoints through ICD-8, ICD-9, and ICD-10

Disease endpoints were defined by clinical expert groups, through International Classification of Diseases (ICD) codes of version 8 (1969-1986), 9 (1987-1995 in Finland, 1987-1996 in Sweden), and 10 (1996-2018 in Finland, 1997-2018 in Sweden).

To remove highly correlated disease diagnoses, we estimated tetrachoric correlations between every two disease endpoints using individual-level data and only kept the one with the highest prevalence if a group of disease diagnoses had tetrachoric correlations higher than 0.7. After removing highly correlated disease diagnoses, we considered 414 disease diagnoses for which we had more than 30 affected individuals in the sibling-based analysis for each sex, in Finland or Sweden.

Disease categories were defined based on the ICD coding system. Among 16 disease categories, infectious-parasitic diseases and congenital anomalies were defined only in Finland while all remaining categories were defined in both countries. Number of diseases endpoints defined for each category is presented in Supplementary **Fig. 2**.

Supplementary **Fig. 2**: Number of disease endpoints by disease category.

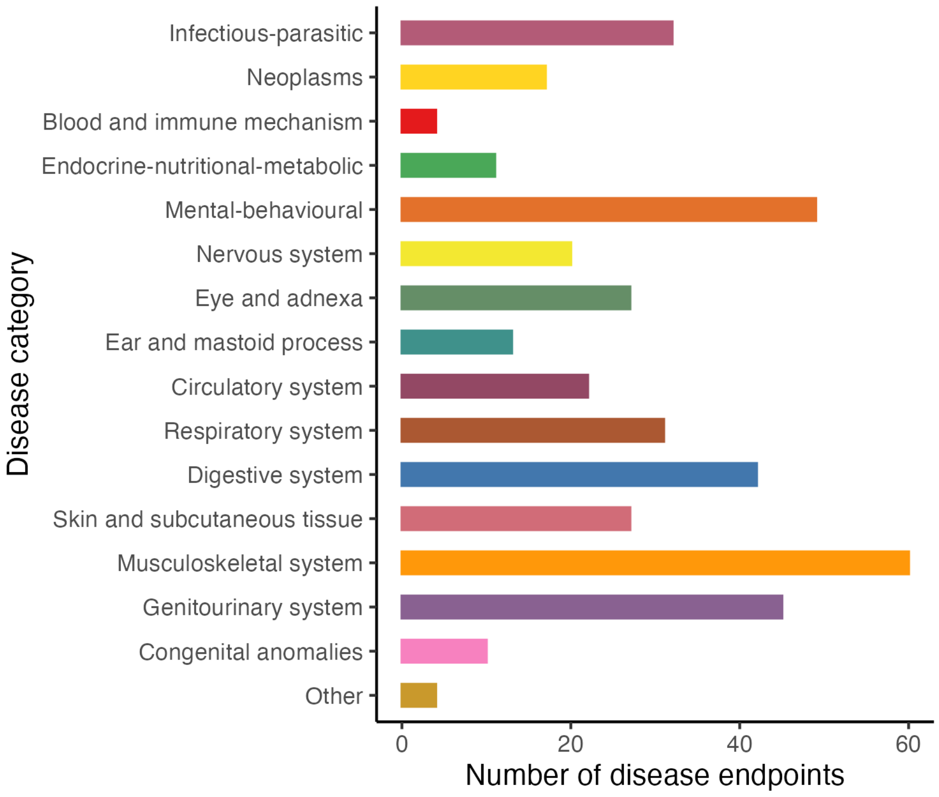

We collected main diagnoses (ICD codes) and admission dates for secondary healthcare inpatient hospital treatments (since 1969, but in Sweden, nationwide coverage began in 1973 for psychiatric diagnoses and 1987 for others63) and specialist outpatient visits in Finland from the Care Register for Health Care (since 1998) and in Sweden from the National Patient Register (since 2001). In Finland, we obtained cancer diagnoses (International Classification of Diseases for Oncology, 3rd Edition) and corresponding dates of diagnoses from the Finnish Cancer Registry, available since 1953. Additionally, for all individuals who died before the end of follow-up, we collected the date of death and basic, immediate, and contributing causes of death from Statistics Finland and Statistics Sweden to capture additional disease diagnoses that were missing from hospital inpatient and outpatient registers. The age of onset was defined as the first record of a disease diagnosis.

Supplementary **Table 5**: Definition of 414 disease endpoints (16 categories) through ICD-8, ICD-9, and ICD-10.

**Disease**	**ICD-8**	**ICD-9**	**ICD-10**	**Category**	**Population**
Respiratory TBC, ICD-8 codes not specifying whether confirmed bacteriologically or histologically	011, 012	--	--	Infectious-parasitic	Finland
Other salmonella infections	003	003	A02	Infectious-parasitic	Finland
Viral and other specified intestinal infections	0088, 0089	008	A08	Infectious-parasitic	Finland
Diarrhoea and gastroenteritis of presumed infectious origin	009	009	A09	Infectious-parasitic	Finland
Tuberculosis	010, 011, 012, 013, 014, 015, 016, 017, 018	010, 011, 012, 013, 014, 015, 016, 017, 018	A15, A16, A17, A18, A19	Infectious-parasitic	Finland
Scarlet fever	0341	0341	A38	Infectious-parasitic	Finland
Meningococcal infection	036	036	A39	Infectious-parasitic	Finland
Other septicaemia	03810, 03820, 03880, 03888, 03897, 03899	0381, 0382, 0383, 0384, 0388	A41	Infectious-parasitic	Finland
Erysipelas	035	035	A46	Infectious-parasitic	Finland
Bacterial infection, other or unspecified	039	040, 041	A48, A49	Infectious-parasitic	Finland
Gonococcal infection	098	098	A54	Infectious-parasitic	Finland
Anogenital herpesviral [herpes simplex] infection	05402	0541A	A60	Infectious-parasitic	Finland
Other predominantly sexually transmitted diseases, not elsewhere classified	0990, 0999	0990A, 0993A, 0994A, 0998X, 0999X	A63, A64	Infectious-parasitic	Finland
Other diseases caused by chlamydiae	--	0769A, 0769B, 0769C, 0769D, 0769E, 0769F, 0769X	A74	Infectious-parasitic	Finland
Acute poliomyelitis	040, 041, 042, 043, 044, 045	045	A80	Infectious-parasitic	Finland
Viral meningitis	045	047	A87	Infectious-parasitic	Finland
Arthropod-borne viral fevers and viral haemorrhagic fevers	061, 067, 068	065, 066, 0786, 0787	A90, A91, A92, A93, A94, A95, A96, A98, A99	Infectious-parasitic	Finland
Herpesviral [herpes simplex] infections	05400, 05401, 05403, 05404, 05405, 05406, 05407, 05409	0540A, 0541B, 0542A, 0542B, 0543A, 0543B, 0544A, 0544B, 0544C, 0544D, 0544X, 0545A, 0546A, 0547A, 0547X, 0548X, 0549X	B00	Infectious-parasitic	Finland
Viral infections characterized by skin and mucous membrane lesions	0790, 0791	0780A, 0781A, 0781B, 0781D, 0783A	B00, B01, B02, B03, B04, B05, B06, B07, B08, B09	Infectious-parasitic	Finland
Varicella [chickenpox]	052	052	B01	Infectious-parasitic	Finland
Zoster [herpes zoster]	053	053	B02	Infectious-parasitic	Finland

Measles	055	055	B05	Infectious-parasitic	Finland
Viral hepatitis	070	070	B15, B16, B17, B18, B19	Infectious-parasitic	Finland
Other viral diseases	075, 0792, 0799	0785, 0787, 0788	B25, B26, B27, B30, B33, B34	Infectious-parasitic	Finland
Mumps	072	072	B26	Infectious-parasitic	Finland
Infectious mononucleosis	075	075	B27	Infectious-parasitic	Finland
Mycoses	110, 111, 112, 113, 114, 115, 116, 117	110, 111, 112, 114, 115, 116, 117, 118	B35, B36, B37, B38, B39, B40, B41, B42, B43, B44, B45, B46, B47, B48, B49	Infectious-parasitic	Finland
Candidiasis	112	112	B37	Infectious-parasitic	Finland
Pediculosis, acariasis and other infestations	132, 133, 134	132, 133, 134	B85, B86, B87, B88, B89	Infectious-parasitic	Finland
Sequelae of other and unspecified infectious and parasitic diseases	066, 079, 074	139	B94	Infectious-parasitic	Finland
Bacterial, viral, and other infectious agents	0340, 074, 078	079, 0340	B95, B96, B97	Infectious-parasitic	Finland
Other infectious diseases	13608, 13609	1368, 1369	B99	Infectious-parasitic	Finland
Malignant neoplasm of testis	186	186	C62	Neoplasms	Finland
Malignant neoplasm of brain	191	191	C71	Neoplasms	Finland
Secondary and unspecified malignant neoplasm of lymph nodes	196	--	C77	Neoplasms	Finland
Lymphoid leukaemia	204	204	C91	Neoplasms	Finland, Sweden
Myeloid leukaemia	205	205	C92	Neoplasms	Finland, Sweden
Carcinoma in situ of uteri, other/unspecified	--	233B	D067, D069	Neoplasms	Sweden
Benign neoplasm: other/unspecified salivary gland	21020	210C	D117, D119	Neoplasms	Sweden
Benign neoplasm: Long bones of lower limb	21306	2137	D162	Neoplasms	Finland
Benign neoplasm: Long bones of lower limb	21306	213H	D162	Neoplasms	Sweden
Benign lipomatous neoplasm of other sites/unspecified	214	214	D177, D179	Neoplasms	Finland
Haemangioma, any site	22700	2280	D180	Neoplasms	Finland
Haemangioma, any site	22700	228A	D180	Neoplasms	Sweden
Melanocytic naevi, other sites/unspecified	--	216W, 216X	D229	Neoplasms	Sweden
Benign neoplasm: Skin of other and unspecified parts of face	21604	2163B, 2163C, 2163D, 2163E, 2163F, 2163G, 2163X	D233	Neoplasms	Finland
Benign neoplasm: Skin of trunk	21606	2165B, 2165C, 2165D, 2165E,	D235	Neoplasms	Finland

		2165F, 2165G, 2165X			
Benign neoplasm: Skin, unspecified	21600, 21609, 2161, 2162, 2168, 2169	2168B, 2168C, 2168D, 2168E, 2168F, 2168G, 2168X, 2169B, 2169C, 2169D, 2169E, 2169F, 2169G, 2169X	D239	Neoplasms	Finland
Leiomyoma of uterus	21899	218	D25	Neoplasms	Finland
Benign neoplasm: Vulva	2212	--	D280	Neoplasms	Finland
Benign neoplasm: Pituitary gland, craniopharyngeal duct	22620	227D	D352, D353	Neoplasms	Sweden
Iron deficiency anaemia secondary to blood loss (chronic)	28000	2800	D500	Blood and immune mechanism	Finland
Iron deficiency anaemia secondary to blood loss (chronic)	28000	280	D500	Blood and immune mechanism	Sweden
Other and unspecified iron deficiency	28008, 28009	2801, 2808, 2809	D501, D508, D509	Blood and immune mechanism	Finland
Other and unspecified iron deficiency	28008, 28009	280	D501, D508, D509	Blood and immune mechanism	Sweden
Allergic purpura	2870	2870	D690	Blood and immune mechanism	Finland
Allergic purpura	2870	287A	D690	Blood and immune mechanism	Sweden
Sarcoidosis	135	135	D86	Blood and immune mechanism	Finland, Sweden
Hypothyroidism, other/unspecified	244	2448X, 2449	E038, E039	Endocrine-nutritional-metabolic	Finland
Hypothyroidism, other/unspecified	244	244W, 244X	E038, E039	Endocrine-nutritional-metabolic	Sweden
Nontoxic multinodular goitre	241	2411	E042	Endocrine-nutritional-metabolic	Finland
Nontoxic multinodular goitre	241	241B	E042	Endocrine-nutritional-metabolic	Sweden
Thyrotoxicosis with diffuse goitre	2420	2420	E050	Endocrine-nutritional-metabolic	Finland
Thyrotoxicosis with diffuse goitre	2420	242A	E050	Endocrine-nutritional-metabolic	Sweden
Thyroiditis, ILD-related definition	245	2448A	E06, E0380	Endocrine-nutritional-metabolic	Finland
Type 1 diabetes	250 (onset before age 40)	2500B, 2501B, 2502B, 2503B, 2504B, 2505B, 2506B, 2507B, 2508B	E10	Endocrine-nutritional-metabolic	Finland
Type 1 diabetes	250 (onset before age 40)	250 (onset before age 40)	E10	Endocrine-nutritional-metabolic	Sweden
Type 2 diabetes	--	2500A, 2501A, 2502A, 2503A, 2504A, 2505A, 2506A, 2507A, 2508A	E11	Endocrine-nutritional-metabolic	Finland
Hypoglycaemia, other or unspecified	25101	251C	E161, E162	Endocrine-nutritional-metabolic	Sweden
Hyperprolactinaemia	--	2531A	E221	Endocrine-nutritional-metabolic	Finland

Polycystic ovarian syndrome	25690	2564	E282	Endocrine-nutritional-metabolic	Finland
Polycystic ovarian syndrome	25690	256E	E282	Endocrine-nutritional-metabolic	Sweden
Other specified/unspecified nutritional deficiencies	26998, 26999	2693, 2698, 2699	E638, E639	Endocrine-nutritional-metabolic	Finland
Other specified/unspecified nutritional deficiencies	26998, 26999	269D, 269W, 269X	E638, E639	Endocrine-nutritional-metabolic	Sweden
Obesity, other/unspecified	27799	2780	E668, E669	Endocrine-nutritional-metabolic	Finland
Obesity, other/unspecified	27799	278A	E668, E669	Endocrine-nutritional-metabolic	Sweden
Other mental disorders due to brain damage and dysfunction and to physical disease	2940, 2941, 2942, 2948, 2949, 309	2938	F06	Mental-behavioural	Finland
Other mental disorders due to brain damage and dysfunction and to physical disease	2940, 2941, 2942, 2948, 2949, 309	293W	F06	Mental-behavioural	Sweden
Acute alcohol intoxication	--	3050	F100	Mental-behavioural	Finland
Acute alcohol intoxication	--	305A	F100	Mental-behavioural	Sweden
Alcohol dependence	303	303	F102	Mental-behavioural	Finland, Sweden
Mental and behavioural disorders due to opioids	3040, 3041	304A	F11	Mental-behavioural	Sweden
Mental and behavioural disorders due to cannabinoids	3045	3052, 3043	F12	Mental-behavioural	Finland
Mental and behavioural disorders due to cannabinoids	3045	304D	F12	Mental-behavioural	Sweden
Mental and behavioural disorders due to sedatives or hypnotics	3042, 3043	3041, 3054	F13	Mental-behavioural	Finland
Mental and behavioural disorders due to sedatives or hypnotics	3042, 3043	304B	F13	Mental-behavioural	Sweden
Mental and behavioural disorders due to cocaine	3044	304C	F14	Mental-behavioural	Sweden
Mental and behavioural disorders due to use of other stimulants, including caffeine	3046	3044, 3057	F15	Mental-behavioural	Finland
Mental and behavioural disorders due to use of other stimulants, including caffeine	3046	304E	F15	Mental-behavioural	Sweden
Mental and behavioural disorders due to hallucinogens	3047	304F	F16	Mental-behavioural	Sweden

Mental and behavioural disorders due to multiple drug use and use of other psychoactive substances	3048, 3049	3049, 3059	F19	Mental-behavioural	Finland
Mental and behavioural disorders due to multiple drug use and use of other psychoactive substances	3048, 3049	304X, 305X	F19	Mental-behavioural	Sweden
Schizophrenia	2950, 2951, 2952, 2953, 2954, 2955, 2956, 2958, 2959	2951, 2952, 2953, 2956, 2959	F20	Mental-behavioural	Finland
Schizophrenia	2950, 2951, 2952, 2953, 2954, 2955, 2956, 2958, 2959	295B, 295C, 295D, 295G, 295X	F20	Mental-behavioural	Sweden
Persistent delusional disorders	297	2971	F22	Mental-behavioural	Finland
Acute and transient psychotic disorders	2980, 2981, 2982, 2983	298	F23	Mental-behavioural	Finland
Schizoaffective disorder	2957	2957	F25	Mental-behavioural	Finland
Schizoaffective disorder	2957	295H	F25	Mental-behavioural	Sweden
Other and unspecified nonorganic psychotic disorders	29999	2989	F28, F29	Mental-behavioural	Finland
Other and unspecified nonorganic psychotic disorders	29999	298X	F28, F29	Mental-behavioural	Sweden
Mood disorders	296, 29800, 3004, 30110	296, 3004A, 3011D	F30, F31, F32, F33, F34, F38, F39	Mental-behavioural	Finland
Bipolar affective disorders	2961, 2962, 2963, 2968	2962, 2963, 2964, 2967	F31	Mental-behavioural	Finland
Bipolar affective disorders	2961, 2962, 2963, 2968	296C, 296D, 296E	F31	Mental-behavioural	Sweden
Persistent mood disorders	3004	3004	F34	Mental-behavioural	Finland
Persistent mood disorders	3004	300E	F34	Mental-behavioural	Sweden
Other and unspecified mood [affective] disorders	29699	--	F38, F39	Mental-behavioural	Sweden
Phobic anxiety disorders	3002	3002C, 3002D, 3002X	F40	Mental-behavioural	Finland
Phobic anxiety disorders	3002	300C	F40	Mental-behavioural	Sweden
Anxiety disorders	3000, 3001, 3002, 30030, 3005, 3006, 3007, 3008, 3009, 305, 30680, 30799	3000, 3001, 3002, 3003, 3006, 3007, 3008, 3009, 3078A, 309	F40, F41, F42, F43, F44, F45, F48	Mental-behavioural	Finland
Panic disorder	--	3000B, 3002B	F410	Mental-behavioural	Finland
Panic disorder	--	300A, 300C	F410	Mental-behavioural	Sweden
Generalized anxiety disorder	--	3000C	F411	Mental-behavioural	Finland
Generalized anxiety disorder	--	300A	F411	Mental-behavioural	Sweden
Other anxiety disorders	3000	3000A	F412, F413, F418, F419	Mental-behavioural	Finland

Other anxiety disorders	3000	300A	F412, F413, F418, F419	Mental-behavioural	Sweden
Obsessive-compulsive disorder	3003	3003	F42	Mental-behavioural	Finland
Obsessive-compulsive disorder	3003	300D	F42	Mental-behavioural	Sweden
Other reaction to severe stress, and adjustment disorders	--	309	F430, F432, F438, F439	Mental-behavioural	Finland, Sweden
Post-traumatic stress disorder	--	309X	F431	Mental-behavioural	Sweden
Dissociative [conversion] disorders	3001	3001	F44	Mental-behavioural	Finland
Dissociative [conversion] disorders	3001	300B	F44	Mental-behavioural	Sweden
Somatoform disorder	305	3007, 3008	F45	Mental-behavioural	Finland
Somatoform disorder	305	300H, 300W	F45	Mental-behavioural	Sweden
Other neurotic disorders	3005, 3006, 3007, 3008, 3009	3006, 3009	F48	Mental-behavioural	Finland
Other neurotic disorders	3005, 3006, 3007, 3008, 3009	300G, 300X	F48	Mental-behavioural	Sweden
Anorexia (incl.atypical)	784	3071, 7830	F500, F501	Mental-behavioural	Finland
Anorexia (incl.atypical)	784	307B, 783A	F500, F501	Mental-behavioural	Sweden
Bulimia nervosa (incl. atypical)	--	307F	F502, F503	Mental-behavioural	Sweden
Other eating disorders	--	307F	F504, F505, F508, F509	Mental-behavioural	Sweden
Nonorganic sleeping disorders	30640	3074	F51	Mental-behavioural	Finland
Paranoid personality disorder	3010	3010	F600	Mental-behavioural	Finland
Schizoid personality disorder	3012	3012	F601	Mental-behavioural	Finland
Dissocial personality disorder	3017	3017	F602	Mental-behavioural	Finland
Emotionally unstable personality disorder	3013	3018D	F603	Mental-behavioural	Finland
Emotionally unstable personality disorder	3013	301W	F603	Mental-behavioural	Sweden
Anxious personality disorder	--	301W	F606	Mental-behavioural	Sweden
Dependent personality disorder	3016	3016	F607	Mental-behavioural	Finland
Other specified and unspecified personality disorders	30188, 3019	3018B, 3018E, 3018X	F608, F609	Mental-behavioural	Finland
Other specified and unspecified personality disorders	30188, 3019	301W	F608, F609	Mental-behavioural	Sweden
Mild intellectual disability	311	3170	F70	Mental-behavioural	Finland
Mild intellectual disability	311	317	F70	Mental-behavioural	Sweden
Moderate intellectual disability	312	3180	F71	Mental-behavioural	Finland

Moderate intellectual disability	312	318A	F71	Mental-behavioural	Sweden
Severe intellectual disability	313	3181	F72	Mental-behavioural	Finland
Severe intellectual disability	313	318B	F72	Mental-behavioural	Sweden
Profound intellectual disability	314	3182	F73	Mental-behavioural	Finland
Profound intellectual disability	314	318C	F73	Mental-behavioural	Sweden
Other and unspecified intellectual disability	315	3199	F78, F79	Mental-behavioural	Finland
Other and unspecified intellectual disability	315	319	F78, F79	Mental-behavioural	Sweden
Specific development disorders of scholastic skills	3061	3150, 3151, 3152	F81	Mental-behavioural	Finland
Behavioural disorders	30899	3120A, 3123D, 3138A	F91	Mental-behavioural	Finland
Other behavioural and emotional disorders with onset usually occurring in childhood and adolescence	3066, 3067	3076, 3077, 3075D, 3075C	F98	Mental-behavioural	Finland
Other behavioural and emotional disorders with onset usually occurring in childhood and adolescence	3066, 3067	307F, 307G, 307H	F98	Mental-behavioural	Sweden
Bacterial meningitis	320	320	G00, G01	Nervous system	Finland, Sweden
Other encephalitis	32308, 32309	3238B, 3238X	G041, G048, G049, G051, G052, G058	Nervous system	Finland
Multiple Sclerosis	34099	340	G35	Nervous system	Finland, Sweden
Epilepsy	3450, 3451, 3453, 3459	3450, 3451, 3454, 3455, 3456, 3457, 3458, 3459	G40	Nervous system	Finland
Epilepsy	3450, 3451, 3453, 3459	345J, 345K, 345L, 345M, 345N, 345W, 345X	G40	Nervous system	Sweden
Status epilepticus	34520	3452, 3453	G41	Nervous system	Finland
Status epilepticus	34520	345P, 345Q	G41	Nervous system	Sweden
Migraine	34609	346	G43	Nervous system	Finland, Sweden
Other headache syndromes	34601	--	G44	Nervous system	Finland, Sweden
Other sleep disorders	--	3478, 3074	G470, G471, G472, G478, G479	Nervous system	Finland
Sleep apnoea	--	3472	G473	Nervous system	Finland
Sleep apnoea	--	347	G473	Nervous system	Sweden
Bell palsy	350	3510	G510	Nervous system	Finland
Bell palsy	350	351A	G510	Nervous system	Sweden
Carpal tunnel syndrome	--	3540	G560	Nervous system	Finland
Carpal tunnel syndrome	--	354A	G560	Nervous system	Sweden
Lesion of radial nerve	35708	3543	G563	Nervous system	Finland
Lesion of sciatic nerve	35399	3550	G570	Nervous system	Finland

Other and unspecified mononeuropathies, also in other diseases	35708	3559	G588, G589, G59	Nervous system	Finland
Muscular dystrophy	33030,33090	3591	G710	Nervous system	Finland
Muscular dystrophy	33030,33090	359B	G710	Nervous system	Sweden
Cerebral palsy	343	343	G80	Nervous system	Finland
Hemiplegia	34400	342	G81	Nervous system	Finland, Sweden
Other and unspecified paralytic syndromes	34408,34409	344	G838, G839	Nervous system	Finland, Sweden
Disorders of brain, other and unspecified	34798,34799	3488,3489	G938, G939, G948	Nervous system	Finland
Disorders of brain, other and unspecified	34798,34799	348W,348X	G938, G939, G948	Nervous system	Sweden
Postprocedural disorders of nervous system	--	3490, 997	G97	Nervous system	Finland
Unilateral visual impairment (ICD-8)	3792, 3793	--	--	Eye and adnexa	Finland
Chalazion	3780	3732	H001	Eye and adnexa	Finland
Disorders of lacrimal system	--	--	H04	Eye and adnexa	Finland
Stenosis and insufficiency of lacrimal passages	36802	3755	H045	Eye and adnexa	Finland
Conjunctivitis (acute, non-atopic)	36000,36008,36009	3720	H100, H102, H103, H105, H108, H109, H131, H132	Eye and adnexa	Finland
Conjunctivitis (acute, non-atopic)	36000,36008,36009	372A	H100, H102, H103, H105, H108, H109, H131, H132	Eye and adnexa	Sweden
Corneal ulcer	36300	3700	H160	Eye and adnexa	Finland
Other keratitis	36301, 36302, 36308, 36309, 36390, 36391, 36392, 36393, 36394, 36398, 36399, 36001, 36002, 36905	3702, 3703, 3705, 3706	H161, H162, H163, H164, H168, H169	Eye and adnexa	Finland
Other keratitis	36301, 36302, 36308, 36309, 36390, 36391, 36392, 36393, 36394, 36398, 36399, 36001, 36002, 36905	370C, 370D, 370F, 370G	H161, H162, H163, H164, H168, H169	Eye and adnexa	Sweden
Keratoconus	37103	371G	H186	Eye and adnexa	Sweden
Acute and subacute iridocyclitis	36400, 36401	3640	H200	Eye and adnexa	Finland
Acute and subacute iridocyclitis	36400, 36401	364A	H200	Eye and adnexa	Sweden
Other cataract	37400, 37401, 37403, 37404, 37405, 37406, 37407, 37408, 37409	3660, 3662, 3663, 3664, 3665, 3668, 3669	H26	Eye and adnexa	Finland
Other cataract	37400, 37401, 37403, 37404, 37405, 37406, 37407, 37408, 37409	366A, 366C, 366D, 366E, 366F, 366W, 366X	H26	Eye and adnexa	Sweden
Aphakia	37872	3793A	H270	Eye and adnexa	Finland
Chorioretinal inflammation	365, 36700	3630, 3631, 3632	H30, H320	Eye and adnexa	Finland
Retinal detachment with retinal break	37602	361A	H330	Eye and adnexa	Sweden
Other, unspecified, and serous retinal detachments	37600, 37601, 37603, 37609	3618, 3619	H332, H335	Eye and adnexa	Finland

Other, unspecified, and serous retinal detachments	37600, 37601, 37603, 37609	361W, 361X	H332, H335	Eye and adnexa	Sweden
Other and unspecified glaucoma	3752, 37598, 37599	3656X, 3656C, 3658, 3659	H408, H409	Eye and adnexa	Finland
Optic neuritis	36702, 36703, 36709	3773	H46	Eye and adnexa	Finland
Convergent concomitant strabismus	37300	3780	H500	Eye and adnexa	Finland
Convergent concomitant strabismus	37300	378A	H500	Eye and adnexa	Sweden
Divergent concomitant strabismus	37301	3781	H501	Eye and adnexa	Finland
Divergent concomitant strabismus	37301	378B	H501	Eye and adnexa	Sweden
Vertical strabismus	37302	3783	H502	Eye and adnexa	Finland
Heterophoria	37303	3784	H505	Eye and adnexa	Finland
Heterophoria	37303	378E	H505	Eye and adnexa	Sweden
Hypermetropia	37001	3670	H520	Eye and adnexa	Finland
Myopia	37002	3671	H521	Eye and adnexa	Finland
Astigmatism	37000	3672	H522	Eye and adnexa	Finland
Anisometropia and aniseikonia	37002	3673	H523	Eye and adnexa	Finland
Presbyopia	37002	3674	H524	Eye and adnexa	Finland
Visual disturbances	--	--	H53	Eye and adnexa	Finland
Amblyopia ex anopsia	37720	3680	H530	Eye and adnexa	Finland
Otitis externa	380	--	H60	Ear and mastoid process	Sweden
Otitis externa, unspecified	38002, 38003, 38004, 38008, 38009	3801A, 3801B	H609	Ear and mastoid process	Finland
Otitis externa, unspecified	38002, 38003, 38004, 38008, 38009	380B	H609	Ear and mastoid process	Sweden
Perichondritis of external ear	38798	3800	H610	Ear and mastoid process	Finland
Nonsuppurative otitis media	--	381	H65	Ear and mastoid process	Finland, Sweden
Acute suppurative otitis media	3810	3820	H660	Ear and mastoid process	Finland
Acute suppurative otitis media	3810	382A	H660	Ear and mastoid process	Sweden
Otitis media, unspecified	3819	--	H669	Ear and mastoid process	Finland
Eustachian salpingitis and obstruction	38400, 38401, 38402	3816	H680, H681	Ear and mastoid process	Finland
Eustachian salpingitis and obstruction	38400, 38401, 38402	381G	H680, H681	Ear and mastoid process	Sweden
Acute mastoiditis	3820, 3830	3830	H700	Ear and mastoid process	Finland
Chronic mastoiditis	3821, 3831	3831	H701	Ear and mastoid process	Finland
Cholesteatoma of middle ear	38700	3853	H71	Ear and mastoid process	Finland
Otosclerosis	38699	387	H80	Ear and mastoid process	Finland
Sensorineural hearing loss	--	3891	H903, H904, H905	Ear and mastoid process	Finland
Sensorineural hearing loss	--	389B	H903, H904, H905	Ear and mastoid process	Sweden

Other specified/unspecified hearing loss	389	3898,3899	H918, H919	Ear and mastoid process	Finland
Other specified/unspecified hearing loss	389	389W,389X	H918, H919	Ear and mastoid process	Sweden
Hypertension, essential	401,402,403,404	401,403	I10	Circulatory system	Finland, Sweden
Hypertensive Renal Disease	40399	4039	I12	Circulatory system	Finland
Angina pectoris	413	413,4110,4111	I20	Circulatory system	Finland
Myocardial infarction, strict	410	410	I21, I22	Circulatory system	Finland, Sweden
Pulmonary embolism	450	415	I26	Circulatory system	Finland, Sweden
Pericarditis	420	420	I30, I32	Circulatory system	Finland, Sweden
Non-rheumatic valve diseases	394, 395, 396, 4240, 4241	424	I34, I35, I36, I37	Circulatory system	Finland, Sweden
Myocarditis	422, 742	422	I40, I41	Circulatory system	Finland, Sweden
Paroxysmal tachycardia	4275	4270, 4271, 4272	I47	Circulatory system	Finland
Paroxysmal tachycardia	4275	427A, 427B, 427C	I47	Circulatory system	Sweden
Atrial fibrillation and flutter	42792	4273	I48	Circulatory system	Finland
Atrial fibrillation and flutter	42792	427D	I48	Circulatory system	Sweden
Other arrhythmias	42790, 42791, 42798, 42799	4274, 4275, 4276, 4278	I49	Circulatory system	Finland
Other arrhythmias	42790, 42791, 42798, 42799	427E, 427F, 427G, 427W	I49	Circulatory system	Sweden
Heart failure, strict	42700, 42710, 428, 7824	4029B, 404, 4148, 428	I50, I110, I130, I132	Circulatory system	Finland
Heart failure, strict	42700, 42710, 428, 7824	402X, 404, 414W, 428	I50, I110, I130, I132	Circulatory system	Sweden
Other or ill-defined heart diseases	42899, 42999	429	I51, I52	Circulatory system	Finland, Sweden
Subarachnoid haemorrhage	430	430	I60	Circulatory system	Finland, Sweden
Intracerebral haemorrhage	431	431	I61	Circulatory system	Sweden
Ischaemic Stroke, excluding all haemorrhages	433, 434, 436	4330A, 4331A, 4339A, 4340, 4341, 4349A, 436	I630, I631, I632, I633, I634, I635, I638, I639, I64	Circulatory system	Finland
Ischaemic Stroke, excluding all haemorrhages	433, 434, 436	433A, 433B, 433X, 434A, 434B, 434X, 436	I630, I631, I632, I633, I634, I635, I638, I639, I64	Circulatory system	Sweden
Aortic aneurysm	441, 930	4411, 4412, 4413, 4414, 4415, 4419, 0930	I711, I712, I713, I714, I715, I716, I718, I719	Circulatory system	Finland
DVT of lower extremities	4510	4511A, 4512A	I802, I803	Circulatory system	Finland
DVT of lower extremities	4510	451B, 451C	I802, I803	Circulatory system	Sweden
Other embolism and thrombosis	453	453	I82	Circulatory system	Finland, Sweden
Varicose veins	454	454	I83	Circulatory system	Finland, Sweden
Varicose veins of other sites	45610, 45698	4563, 4564, 4565, 4566, 4568	I86	Circulatory system	Finland
Varicose veins of other sites	45610, 45698	456D, 456E, 456F, 456G, 456W	I86	Circulatory system	Sweden

Nonspecific lymphadenitis	2891, 2892, 2893	2891, 2892	I88	Circulatory system	Finland
Nonspecific lymphadenitis	2891, 2892, 2893	289B, 289C	I88	Circulatory system	Sweden
Acute nasopharyngitis (common cold)	460	460	J00	Respiratory system	Finland, Sweden
Acute sinusitis	461	461	J01	Respiratory system	Finland, Sweden
Acute pharyngitis	462	462	J02	Respiratory system	Finland, Sweden
Other and unspecified tonsillitis	463	463	J03	Respiratory system	Finland, Sweden
Acute laryngitis and tracheitis	46401, 46403, 46409, 50803	4640, 4641, 4642, 4644	J04, J050	Respiratory system	Finland
Acute laryngitis and tracheitis	46401, 46402, 46409, 50800	464A, 464B, 464C, 464E	J04, J050	Respiratory system	Sweden
Acute epiglottitis	46403	464D	J051	Respiratory system	Sweden
Acute upper respiratory infections of multiple and unspecified sites	465	465	J06	Respiratory system	Finland, Sweden
All influenza	470, 472, 473, 474	487	J101, J108, J111, J118	Respiratory system	Finland, Sweden
Viral pneumonia (unknown virus, not influenza)	480	480	J128, J129	Respiratory system	Sweden
Pneumonia due to Streptococcus pneumoniae	48199	481	J13	Respiratory system	Finland, Sweden
Bacterial pneumonia (organism specified)	48199, 482	481, 482, 483	J13, J14, J15, J16, J17	Respiratory system	Finland
Pneumonia due to other infectious organisms, not elsewhere classified	48399	483	J16, J172, J173, J178	Respiratory system	Finland, Sweden
Pneumonia, organism unspecified	48502, 48509, 486	485	J18	Respiratory system	Finland, Sweden
Acute bronchitis	--	4660	J20	Respiratory system	Finland
Acute bronchitis	--	466A	J20	Respiratory system	Sweden
Unspecified acute lower respiratory infection	46699	--	J22	Respiratory system	Finland, Sweden
Vasomotor and allergic rhinitis	507	477	J30	Respiratory system	Finland, Sweden
Chronic rhinitis, nasopharyngitis and pharyngitis	502	472	J31	Respiratory system	Finland
Chronic sinusitis	503	473	J32	Respiratory system	Finland, Sweden
Nasal polyp	505	471	J33	Respiratory system	Finland, Sweden
Diseases of vocal cords and larynx, other diseases of upper respiratory tract, not elsewhere classified	50800, 50801, 50802, 50804, 50805, 50806, 50808, 50809	478	J34, J38, J39	Respiratory system	Finland, Sweden
Deviated nasal septum	50499	470	J342	Respiratory system	Finland, Sweden
Chronic diseases of tonsils and adenoids	500	474	J35	Respiratory system	Finland, Sweden
Peritonsillar abscess	501	475	J36	Respiratory system	Finland, Sweden

Bronchitis, not specified as acute or chronic	49099	490	J40	Respiratory system	Finland, Sweden
Unspecified chronic bronchitis	49109	4919	J42	Respiratory system	Finland
Non-allergic asthma (mode)	--	--	J451	Respiratory system	Finland
Bronchiectasis	51899	494	J47	Respiratory system	Finland
Other interstitial pulmonary diseases	48499, 51701	515, 516	J84	Respiratory system	Finland
Pneumothorax	51299	512	J93	Respiratory system	Finland, Sweden
Other pleural conditions	51101, 51109	511A, 511W, 511X	J94	Respiratory system	Sweden
Other respiratory disorders and diseases	51900, 51901, 51921, 51922, 51925, 51928, 51991, 51992, 51993, 51994, 51998	5180, 5181, 5188, 519	J95, J96, J98	Respiratory system	Finland
Other respiratory disorders and diseases	51900, 51901, 51921, 51922, 51925, 51928, 51991, 51992, 51993, 51994, 51998	518A,518B,518W,519	J95, J96, J98	Respiratory system	Sweden
Oesophagitis	5301	530B	K20	Digestive system	Sweden
Gastro-oesophageal reflux disease	--	5301A	K21	Digestive system	Finland
Gastro-oesophageal reflux disease	--	530B	K21	Digestive system	Sweden
Other diseases of oesophagus	5302, 5309	5305, 5306, 5307, 5308, 5309	K224, K225, K226, K228, K229	Digestive system	Finland
Other diseases of oesophagus	5302, 5309	530F, 530G, 530H, 530W, 530X	K224, K225, K226, K228, K229	Digestive system	Sweden
Barret oesophagus	--	530B	K227	Digestive system	Sweden
Duodenal ulcer	--	532	K26	Digestive system	Finland
Acute gastritis	53500, 53501, 53502	5350	K290, K291	Digestive system	Finland
Acute gastritis	53500, 53501, 53502	535A	K290, K291	Digestive system	Sweden
Alcoholic gastritis	--	5353	K292	Digestive system	Finland
Chronic gastritis	53503	5351, 5354	K293, K294, K295	Digestive system	Finland
Other gastritis (incl. Duodenitis)	53504, 53505, 53506, 53507, 53508, 53509	5352, 5355, 5356	K296, K297, K298, K299	Digestive system	Finland
Other gastritis (incl. Duodenitis)	53504, 53505, 53506, 53507, 53508, 53509	535C, 535F, 535G	K296, K297, K298, K299	Digestive system	Sweden
Functional dyspepsia	5361	5368	K30	Digestive system	Finland
Functional dyspepsia	5361	536W	K30	Digestive system	Sweden
Other diseases of stomach and duodenum	5360, 5369, 537	5361, 5362, 5368X, 5369, 537	K31	Digestive system	Finland
Acute appendicitis	540	540	K35	Digestive system	Finland, Sweden
Other appendicitis	541, 542	541, 542	K36, K37	Digestive system	Finland, Sweden
Other diseases of appendix	543	543	K38	Digestive system	Finland
Inguinal hernia	550	550	K40	Digestive system	Finland, Sweden
Umbilical hernia	5511	5511, 5521, 5531	K42	Digestive system	Finland
Umbilical hernia	5511	551B, 552B, 553B	K42	Digestive system	Sweden
Ventral hernia	5512	5512, 5522, 5532	K43	Digestive system	Finland
Ventral hernia	5512	551C, 552C, 553C	K43	Digestive system	Sweden
Diaphragmatic hernia	5513	551D, 552D, 553D	K44	Digestive system	Sweden

Ulcerative colitis	5631,569	5560A, 5561A, 5562A, 5563A, 5568X	K51	Digestive system	Finland
Ulcerative colitis	5631,569	556	K51	Digestive system	Sweden
Other noninfective gastroenteritis and colitis	562,5639	558, 5564	K52	Digestive system	Finland
Other noninfective gastroenteritis and colitis	562,5639	558,556	K52	Digestive system	Sweden
Paralytic ileus	5601	560B	K560	Digestive system	Sweden
Other or unspecified ileus, impaction, or obstruction	5609	5608X, 5609X	K564, K566, K567	Digestive system	Finland
Other or unspecified ileus, impaction, or obstruction	5609	560W, 560X	K564, K566, K567	Digestive system	Sweden
Intestinal adhesions without obstruction	56095, 56096	5608A	K565	Digestive system	Finland
Intestinal adhesions without obstruction	56095, 56096	560W	K565	Digestive system	Sweden
Irritable bowel syndrome	56419	5641	K58	Digestive system	Finland
Irritable bowel syndrome	56419	564B	K58	Digestive system	Sweden
Other functional intestinal disorders	56400, 56410, 56411, 56490, 56498, 56499	5640, 5642, 5645, 5646, 5647, 5648, 5649	K59	Digestive system	Finland
Other functional intestinal disorders	56400, 56410, 56411, 56490, 56498, 56499	564A, 564C, 564F, 564G, 564H, 564W, 564X	K59	Digestive system	Sweden
Fissure and fistula of anal and rectal regions	565	565	K60	Digestive system	Finland, Sweden
Abscess of anal and rectal regions	566	566	K61	Digestive system	Finland, Sweden
Other diseases of anus and rectum	56906, 56907	5690, 5691, 5692, 5694	K62	Digestive system	Finland
Other diseases of anus and rectum	56906, 56907	569A, 569B, 569C, 569E	K62	Digestive system	Sweden
Other diseases of intestine	56900, 56901, 56902, 56903, 56904, 56905, 56908, 56909	5695, 5696, 5698, 5699	K63	Digestive system	Finland
Other diseases of intestine	56900, 56901, 56902, 56903, 56904, 56905, 56908, 56909	569F, 569G, 569W, 569X	K63	Digestive system	Sweden
Haemorrhoids and perianal venous thrombosis	455	455	K64	Digestive system	Finland, Sweden
Acute peritonitis	567	567	K65	Digestive system	Finland, Sweden
Other diseases of peritoneum	56900, 56901, 56903, 56904, 56905, 56906, 56907, 56908, 56909	568	K66	Digestive system	Finland, Sweden
Alcoholic liver disease	5710	5710, 5711, 5712, 5713	K70	Digestive system	Finland
Other inflammatory liver diseases	57300	5720	K75	Digestive system	Finland
Other diseases of liver	57301, 57302, 57303, 57308, 57309	5718, 5719, 5721, 5722, 5723, 5724, 5728	K76	Digestive system	Finland

Other diseases of liver	57301, 57302, 57303, 57308, 57309	571W, 571X, 572B, 572C, 572D, 572E, 572W	K76	Digestive system	Sweden
Cholelithiasis	574	574	K80	Digestive system	Finland, Sweden
Cholecystitis	57500, 57501, 57502, 57503	5750, 5751	K81	Digestive system	Finland
Cholecystitis	57500, 57501, 57502, 57503	575A, 575B	K81	Digestive system	Sweden
Acute pancreatitis	5770	5770	K85	Digestive system	Finland
Alcohol-induced acute pancreatitis	--	5770D, 5770E	K852	Digestive system	Finland
Alcohol-induced chronic pancreatitis	--	5771C,5771D	K860	Digestive system	Finland
Coeliac disease	--	5790	K900	Digestive system	Finland
Intestinal malabsorption	2691	5791, 5792, 5794, 5798	K901, K902, K903, K904, K908, K909	Digestive system	Finland
Intestinal malabsorption	2691	579B, 579C, 579E, 579W	K901, K902, K903, K904, K908, K909	Digestive system	Sweden
Impetigo	684,70708	684	L01	Skin and subcutaneous tissue	Finland
Cutaneous abscess, furuncle, and carbuncle	680	680	L02	Skin and subcutaneous tissue	Finland, Sweden
Cellulitis	681,682	681,682	L03	Skin and subcutaneous tissue	Finland, Sweden
Acute lymphadenitis	683	683	L04	Skin and subcutaneous tissue	Finland, Sweden
Pilonidal cyst	--	685	L05	Skin and subcutaneous tissue	Finland, Sweden
Pyoderma	68600	6860	L080	Skin and subcutaneous tissue	Finland
Other and unspecified local infections of skin and subcutaneous tissue	68690, 68691, 68698	6868, 6869	L088, L089	Skin and subcutaneous tissue	Finland
Atopic dermatitis	691	6918A, 6918B, 6918C	L20	Skin and subcutaneous tissue	Finland
Atopic dermatitis	691	691W	L20	Skin and subcutaneous tissue	Sweden
Seborrhoeic dermatitis	690, 70630	690	L21	Skin and subcutaneous tissue	Finland
Allergic contact dermatitis	--	6920B, 6921B, 6922B, 6924B, 6924D, 6924H, 6924L, 6925B, 6925D, 6926B, 6926D	L23	Skin and subcutaneous tissue	Finland
Other contact dermatitis	6921, 6922, 6923, 6924, 6926, 69281	6920A, 6921A, 6922A, 6923, 6924A, 6924C, 6924E, 6924F, 6924G, 6924K, 6924M, 6924N, 6925A, 6925C, 6925X, 6926A, 6926C, 6926X, 6927, 6928, 6929	L24, L25	Skin and subcutaneous tissue	Finland
Pruritus	6980, 6981, 6989	698A, 698B, 698W, 698X	L29	Skin and subcutaneous tissue	Sweden
Other and unspecified dermatitis	69282, 69288, 6929	--	L308, L309	Skin and subcutaneous tissue	Finland, Sweden

Psoriasis vulgaris	--	6961A	L400	Skin and subcutaneous tissue	Finland
Psoriasis vulgaris	--	696B	L400	Skin and subcutaneous tissue	Sweden
Other and unspecified psoriasis	69619	6968X	L408, L409	Skin and subcutaneous tissue	Finland
Other and unspecified psoriasis	69619	696W	L408, L409	Skin and subcutaneous tissue	Sweden
Allergic urticaria	--	7080	L500	Skin and subcutaneous tissue	Finland
Allergic urticaria	--	708A	L500	Skin and subcutaneous tissue	Sweden
Other and unspecified urticaria	708	7088, 7089	L508, L509	Skin and subcutaneous tissue	Finland
Other and unspecified urticaria	708	708W, 708X	L508, L509	Skin and subcutaneous tissue	Sweden
Erythema nodosum	69520	6952	L52	Skin and subcutaneous tissue	Finland
Other and unspecified acute skin changes due to ultraviolet radiation	6928	6927B, 6927C, 6927D, 6927E, 6927H, 6927X	L568, L569	Skin and subcutaneous tissue	Finland
Ingrowing nail	70302	7030	L600	Skin and subcutaneous tissue	Finland
Acne vulgaris	70610	7061A	L700	Skin and subcutaneous tissue	Finland
Acne vulgaris	70610	706B	L700	Skin and subcutaneous tissue	Sweden
Rosacea	--	--	L71	Skin and subcutaneous tissue	Sweden
Follicular cysts of skin and subcutaneous tissue	70620	7062A	L72	Skin and subcutaneous tissue	Finland
Follicular cysts of skin and subcutaneous tissue	70620	706C	L72	Skin and subcutaneous tissue	Sweden
Corns and callosities	700	700	L84	Skin and subcutaneous tissue	Finland
Scar conditions and fibrosis of skin	70906,70907	709C	L905	Skin and subcutaneous tissue	Sweden
Hypertrophic scar	70130	7014B	L910	Skin and subcutaneous tissue	Finland
Hypertrophic scar	70130	701E	L910	Skin and subcutaneous tissue	Sweden
Other and unspecified disorders of skin and subcutaneous tissue	70198, 70908, 70909	7019X, 7098C, 7098X, 7099	L988, L989	Skin and subcutaneous tissue	Finland
Pyogenic arthritis	710	7110, 7119	M00, M01	Musculoskeletal system	Finland
Pyogenic arthritis	710	711A, 711X	M00, M01	Musculoskeletal system	Sweden
Reactive arthropathies	71490	7111, 7115, 7118, 713	M02, M03	Musculoskeletal system	Finland
Reactive arthropathies	71490	711B, 711F, 711W, 713	M02, M03	Musculoskeletal system	Sweden
Polyarthropathies	--	--	M05, M06, M07, M08, M09, M10, M11, M12, M13, M14	Musculoskeletal system	Finland, Sweden
Other/unspecified seropositive rheumatoid arthritis	--	7140A	M058, M059	Musculoskeletal system	Finland

Seronegative rheumatoid arthritis	--	7140B	M060	Musculoskeletal system	Finland
Juvenile idiopathic arthritis	7120	7143A	M080	Musculoskeletal system	Finland
Juvenile idiopathic arthritis	7120	714D	M080	Musculoskeletal system	Sweden
Other juvenile arthritis	--	7143B, 7143C, 7143D, 7143X	M081, M082, M083, M084, M088, M089, M09	Musculoskeletal system	Finland
Monoarthritis, not elsewhere classified	71100, 71101, 71102, 71103, 71104, 71105, 71106	7166	M131	Musculoskeletal system	Finland
Monoarthritis, not elsewhere classified	71100, 71101, 71102, 71103, 71104, 71105, 71106	716G	M131	Musculoskeletal system	Sweden
Other specific/unspecified arthritis	71108, 71109, 71498	7169	M138, M139	Musculoskeletal system	Finland
Coxarthrosis	71300	715B,715C	M16	Musculoskeletal system	Sweden
Gonarthrosis	71301	7151F,7152F	M17	Musculoskeletal system	Finland
Gonarthrosis	71301	715B,715C	M17	Musculoskeletal system	Sweden
Other arthrosis	71302, 71308, 71309, 71320	7151A, 7151B, 7151C, 7151D, 7151G, 7151H, 7151K, 7151X, 7152A, 7152B, 7152C, 7152D, 7152G, 7152H, 7152K, 7152X	M18, M19	Musculoskeletal system	Finland
Other arthrosis	71302, 71308, 71309, 71320	715B, 715C	M18, M19	Musculoskeletal system	Sweden
Other joint disorders	--	--	M20, M21, M22, M23, M24, M25	Musculoskeletal system	Finland, Sweden
Hallux valgus (acquired)	737, 73099	7350, 7271	M201	Musculoskeletal system	Finland
Hallux valgus (acquired)	737, 73099	735A, 727B	M201	Musculoskeletal system	Sweden
Flat foot [pes planus] (acquired)	736	734	M214	Musculoskeletal system	Finland
Other specified acquired deformities of limbs	73808, 73809	7368,7369	M218, M219	Musculoskeletal system	Finland
Other disorders of patella	--	7177	M222, M223, M224, M228, M229	Musculoskeletal system	Finland
Other disorders of patella	--	717H	M222, M223, M224, M228, M229	Musculoskeletal system	Sweden
Meniscus derangement	--	7170, 7174, 7175	M230, M231, M232, M233	Musculoskeletal system	Finland
Meniscus derangement	--	717A, 717E,7 17F	M230, M231, M232, M233	Musculoskeletal system	Sweden
Internal derangement of knee	72410	7176, 7178, 7179	M234, M235, M236, M238, M239	Musculoskeletal system	Finland
Internal derangement of knee	72410	717G, 717W, 717X	M234, M235, M236, M238, M239	Musculoskeletal system	Sweden
Loose body in joint	72901	7181	M240	Musculoskeletal system	Finland
Loose body in joint	72901	718B	M240	Musculoskeletal system	Sweden
Other articular cartilage disorders	7249	7180	M241	Musculoskeletal system	Finland
Other articular cartilage disorders	7249	718A	M241	Musculoskeletal system	Sweden

Other specific joint derangements/joint disorders	72902, 72903	7188, 7189	M242, M248, M249, M25	Musculoskeletal system	Finland
Other specific joint derangements/joint disorders	72902, 72903	718W, 718X	M242, M248, M249, M25	Musculoskeletal system	Sweden
Pathological/recurrent dislocation and subluxation of joint, not elsewhere classified	72904	7182	M243	Musculoskeletal system	Finland
Pathological/recurrent dislocation and subluxation of joint, not elsewhere classified	72904	718C	M243	Musculoskeletal system	Sweden
Recurrent dislocation and subluxation of joint	--	7183	M244	Musculoskeletal system	Finland
Recurrent dislocation and subluxation of joint	--	718D	M244	Musculoskeletal system	Sweden
Systemic lupus erythematosus	7431	7100	M32	Musculoskeletal system	Finland
Systemic lupus erythematosus	7431	710A	M32	Musculoskeletal system	Sweden
Other specified/unspecified systemic involvement of connective tissue	73491, 73498, 73499	710W,710X	M358, M359	Musculoskeletal system	Sweden
Torticollis	7172	7235	M436	Musculoskeletal system	Finland
Other specified/unspecified deforming dorsopathies	735	7328, 7329, 7378, 7379	M438, M439	Musculoskeletal system	Finland
Other specified/unspecified deforming dorsopathies	735	732W, 732X, 737W, 737X	M438, M439	Musculoskeletal system	Sweden
Ankylosing spondylitis	7124	7200	M45	Musculoskeletal system	Finland
Ankylosing spondylitis	7124	720A	M45	Musculoskeletal system	Sweden
Spondyloarthritis	7124, 7131, 72699	720	M45, M46	Musculoskeletal system	Finland
Spondylopathies	--	--	M45, M46, M47, M48, M49	Musculoskeletal system	Finland, Sweden
Cervical disc disorders	7250	7220, 7224, 7227, 7228A	M50	Musculoskeletal system	Finland
Other intervertebral disc disorders	72510, 72519, 72588, 72599	7221, 7223, 7225, 7226, 7229	M51	Musculoskeletal system	Finland
Other intervertebral disc disorders	72510, 72519, 72588, 72599	722B, 722D, 722F, 722G, 722X	M51	Musculoskeletal system	Sweden
Radiculopathy	7283, 7288	7234, 7244	M541	Musculoskeletal system	Finland
Radiculopathy	7283, 7288	723E, 724E	M541	Musculoskeletal system	Sweden
Cervicalgia	7289	7231	M542	Musculoskeletal system	Finland
Cervicalgia	7289	723B	M542	Musculoskeletal system	Sweden
Sciatica, lumbago	7170	--	M543, M544	Musculoskeletal system	Finland, Sweden
Low back pain	72870	7242	M545	Musculoskeletal system	Finland
Low back pain	72870	724C	M545	Musculoskeletal system	Sweden
Pain in thoracic spine	7285	7241	M546	Musculoskeletal system	Finland

Other/unspecified dorsalgia	7289	7245	M548, M549	Musculoskeletal system	Finland
Other/unspecified dorsalgia	7289	724F	M548, M549	Musculoskeletal system	Sweden
Other specified disorders of muscle	73390	7288,7289	M628, M629	Musculoskeletal system	Finland
Disorders of synovium and tendon	--	--	M65, M66, M67, M68	Musculoskeletal system	Finland, Sweden
Radial styloid tenosynovitis [de Quervain]	--	727A	M654	Musculoskeletal system	Sweden
Other/unspecified synovitis and tenosynovitis	73100, 73101, 73102, 73103	7270B, 7270C, 7270X	M658, M659	Musculoskeletal system	Finland
Other/unspecified synovitis and tenosynovitis	73100, 73101, 73102, 73103	727A	M658, M659	Musculoskeletal system	Sweden
Spontaneous rupture of synovium and tendon	--	7276	M66	Musculoskeletal system	Finland
Ganglion	--	7274	M674	Musculoskeletal system	Finland
Ganglion	--	727E	M674	Musculoskeletal system	Sweden
Prepatellar bursitis	--	7266E	M704	Musculoskeletal system	Finland
Other bursal cyst	--	7274	M713	Musculoskeletal system	Finland
Other bursitis, not elsewhere classified	73105, 73109	7273	M715	Musculoskeletal system	Finland
Other bursitis, not elsewhere classified	73105, 73109	727D	M715	Musculoskeletal system	Sweden
Rotator cuff syndrome	--	7261X	M751	Musculoskeletal system	Finland
Achilles tendinitis	--	7267A	M766	Musculoskeletal system	Finland
Other/unspecified enthesopathies, not elsewhere classified	73105, 73109	7268, 7269	M778, M779	Musculoskeletal system	Finland
Other/unspecified enthesopathies, not elsewhere classified	73105, 73109	726W, 726X	M778, M779	Musculoskeletal system	Sweden
Rheumatism, unspecified	71899	7290	M790	Musculoskeletal system	Finland
Myalgia	--	729B	M791	Musculoskeletal system	Sweden
Pain in limb	--	--	M796	Musculoskeletal system	Finland, Sweden
Other specified/unspecified soft tissue disorders	71798	729W, 729X	M798, M799	Musculoskeletal system	Sweden
Osteomyelitis	720	7300, 7301, 7302, 7303, 7309	M86	Musculoskeletal system	Finland
Osteomyelitis	720	730A, 730B, 730C, 730D, 730X	M86	Musculoskeletal system	Sweden
Other specified/unspecified disorders of bone/cartilage	72398, 72399	7339	M898, M899, M948, M949	Musculoskeletal system	Finland
Other specified/unspecified disorders of bone/cartilage	72398, 72399	733X	M898, M899, M948, M949	Musculoskeletal system	Sweden
Juvenile osteochondrosis	72209, 72228, 72229	7321X, 7323, 7324, 7325, 7326	M91, M92	Musculoskeletal system	Finland
Other osteochondropathies	72200, 72210, 72211, 72219, 72220, 72221,	7322, 7327, 7328, 7329	M93	Musculoskeletal system	Finland

	72222, 72223, 72288, 72299				
Other osteochondropathies	72200, 72210, 72211, 72219, 72220, 72221, 72222, 72223, 72288, 72299	732C, 732H, 732W, 732X	M93	Musculoskeletal system	Sweden
Other acquired deformities of musculoskeletal system and connective tissue	73808, 73809	738	M95	Musculoskeletal system	Finland, Sweden
Acute nephritic syndrome	580	5800, 5808, 5809	N00	Genitourinary system	Finland
Acute nephritic syndrome	580	580A, 580W, 580X	N00	Genitourinary system	Sweden
Chronic nephritic syndrome	582	582	N03	Genitourinary system	Finland, Sweden
Unspecified nephritic syndrome	583	583	N05	Genitourinary system	Finland
Acute tubulo-interstitial nephritis	5901	5901	N10	Genitourinary system	Finland
Acute tubulo-interstitial nephritis	5901	590B	N10	Genitourinary system	Sweden
Chronic tubulo-interstitial nephritis	5900	5900	N11	Genitourinary system	Finland
Chronic tubulo-interstitial nephritis	5900	590A	N11	Genitourinary system	Sweden
Tubulo-interstitial nephritis, not specified as acute or chronic	5909	5909	N12	Genitourinary system	Finland
Hydronephrosis	591	591	N130, N131, N132, N133	Genitourinary system	Finland, Sweden
Other obstructive and reflux uropathy	5933, 5934, 5935	5933, 5934, 5935, 5937	N134, N135, N136, N137, N138, N139	Genitourinary system	Finland
Other obstructive and reflux uropathy	5933, 5934, 5935	593D, 593E, 593F, 593H	N134, N135, N136, N137, N138, N139	Genitourinary system	Sweden
Other renal tubulo-interstitial diseases	5902, 5909	5902,5909	N15	Genitourinary system	Finland
Calculus of kidney and ureter	592	592	N20	Genitourinary system	Finland, Sweden
Other specified disorders of kidney and ureter	59322	5930, 5931, 5938A, 5938C, 5938X	N280, N288, N289	Genitourinary system	Finland
Cystitis	595	595	N30	Genitourinary system	Finland, Sweden
Other disorders of bladder	596	596F, 596W, 596X	N32	Genitourinary system	Sweden
Urethritis and urethral syndrome	597	--	N34	Genitourinary system	Finland, Sweden
Urethral stricture	598	--	N35	Genitourinary system	Finland, Sweden
Other disorders of urethra and urinary system	599	5938B, 5965C, 5965D, 5965E, 5965F, 5965X, 6256	N36, N39	Genitourinary system	Finland
Other disorders of urethra and urinary system	599	593W, 596F, 599, 625G	N36, N39	Genitourinary system	Sweden
Inflammatory diseases of prostate (prostatitis)	601	601	N41	Genitourinary system	Finland, Sweden
Hydrocele	603	603	N430, N431, N432, N433	Genitourinary system	Finland, Sweden

Spermatocele	6076	6081	N434	Genitourinary system	Finland
Torsion of testis	6077	6082	N44	Genitourinary system	Finland
Torsion of testis	6077	608C	N44	Genitourinary system	Sweden
Orchitis and epididymitis	604	604	N45	Genitourinary system	Finland, Sweden
Redundant prepuce, phimosis and paraphimosis	605	605	N47	Genitourinary system	Finland, Sweden
Other disorders of penis	6070, 6071, 6072, 6073, 60790	6070A, 6071A, 6071B, 6071X, 6072A, 6073A, 6078A, 6078X, 6079X	N480, N481, N482, N483, N485, N486, N488, N489	Genitourinary system	Finland
Other disorders of penis	6070, 6071, 6072, 6073, 60790	607A, 607B, 607C, 607D, 607W, 607X	N480, N481, N482, N483, N485, N486, N488, N489	Genitourinary system	Sweden
Other disorders of male genital organs	60780, 60791, 60798	6083, 6088	N50	Genitourinary system	Finland
Other disorders of male genital organs	60780, 60791, 60798	608D, 608W	N50	Genitourinary system	Sweden
Benign mammary dysplasia	610	610	N60	Genitourinary system	Finland, Sweden
Hypertrophy of breast	6111	6111	N62	Genitourinary system	Finland
Hypertrophy of breast	6111	611B	N62	Genitourinary system	Sweden
Unspecified lump in breast	--	6117B	N63	Genitourinary system	Finland
Other disorders of breast	6112, 6119	6112, 6113, 6114, 6115, 6116, 6117A, 6117C, 6117D, 6117E, 6118, 6119	N64	Genitourinary system	Finland
Other disorders of breast	6112, 6119	611C, 611D, 611E, 611F, 611G, 611H, 611W, 611X	N64	Genitourinary system	Sweden
Salpingitis and oophoritis	612, 613, 614	6140, 6141, 6142	N70	Genitourinary system	Finland
Salpingitis and oophoritis	612, 613, 614	614A, 614B, 614C	N70	Genitourinary system	Sweden
Cyst of Bartholin Gland	--	6162	N750	Genitourinary system	Finland
Other and unspecified disease of Bartholin gland	62212, 62213	--	N758, N759	Genitourinary system	Finland
Vaginitis/vulvovaginitis/vulvitis/abscess of vulva	6221	6161	N760, N761, N762, N763, N764	Genitourinary system	Finland
Vaginitis/vulvovaginitis/vulvitis/abscess of vulva	6221	616B	N760, N761, N762, N763, N764	Genitourinary system	Sweden
Other inflammation of vagina/vulva	62218, 62219	6168, 6169	N768	Genitourinary system	Finland
Other inflammation of vagina/vulva	62218, 62219	616W, 616X	N768	Genitourinary system	Sweden
Vulvovaginal ulceration/inflammation in other diseases	--	--	N77	Genitourinary system	Finland
Torsion of ovary, ovarian pedicle, and fallopian tube	6150	6205	N835	Genitourinary system	Finland
Torsion of ovary, ovarian pedicle, and fallopian tube	6150	620F	N835	Genitourinary system	Sweden

Polyp of the female genital tract	62520	6210, 6227, 6237, 6246	N84	Genitourinary system	Finland
Polyp of the female genital tract	62520	621A, 622H, 623H, 624G	N84	Genitourinary system	Sweden
Erosion and ectropion of cervix uteri	62191	6220A	N86	Genitourinary system	Finland
Erosion and ectropion of cervix uteri	62191	622A	N86	Genitourinary system	Sweden
Other noninflammatory disorders of cervix uteri	62191	6222, 6223, 6224, 6225, 6226, 6228, 6229	N88	Genitourinary system	Finland
Other noninflammatory disorders of cervix uteri	62191	622C, 622D, 622E, 622F, 622G, 622W, 622X	N88	Genitourinary system	Sweden
Other noninflammatory disorders of vagina	6200, 6210, 6293, 62950, 6297	623	N89	Genitourinary system	Finland, Sweden
Other noninflammatory disorders of vulva and perineum	62911, 62920	624	N90	Genitourinary system	Finland
Amenorrhoea	6260	6260	N910, N911, N912	Genitourinary system	Finland
Amenorrhoea	6260	626A	N910, N911, N912	Genitourinary system	Sweden
Oligomenorrhoea	6261	6261	N913, N914, N915	Genitourinary system	Finland
Oligomenorrhoea	6261	626B	N913, N914, N915	Genitourinary system	Sweden
Excessive, frequent, and irregular menstruation	6262, 6264	6262, 6263, 6264, 6265, 6266, 6270	N92	Genitourinary system	Finland
Excessive, frequent, and irregular menstruation	6262,6264	626C, 626D, 626E, 626F, 626G, 627A	N92	Genitourinary system	Sweden
Other abnormal uterine and vaginal bleeding	6265, 6266, 6267, 6269	6267, 6268, 6269	N93	Genitourinary system	Finland
Other abnormal uterine and vaginal bleeding	6265, 6266, 6267 ,6269	626H, 626W, 626X	N93	Genitourinary system	Sweden
Pain and other conditions associated with female genital organs and menstrual cycle	6263	6250, 6251, 6252, 6253, 6254, 6255, 6258, 6259	N94	Genitourinary system	Finland
Pain and other conditions associated with female genital organs and menstrual cycle	6263	625A, 625B, 625C, 625D, 625E, 625F, 625W, 625X	N94	Genitourinary system	Sweden
Congenital hydrocephalus	742	7423	Q03	Congenital anomalies	Finland
Congenital malformations of eye, ear, face, and neck	744, 745	743, 744	Q10, Q11, Q12, Q13, Q14, Q15, Q16, Q17, Q18	Congenital anomalies	Finland
Congenital anomalies of heart	746	745,746	Q20, Q21, Q22, Q23, Q24	Congenital anomalies	Finland
Congenital malformations of the circulatory system	746, 747	745, 746, 747	Q20, Q21, Q22, Q23, Q24, Q25, Q26, Q27, Q28	Congenital anomalies	Finland
Congenital anomalies of digestive system	749, 750, 751	749, 750, 751	Q35, Q36, Q37, Q38, Q39, Q40, Q41, Q42, Q43, Q44, Q45	Congenital anomalies	Finland

Other congenital malformations of the digestive system	750, 751	750, 751	Q38, Q39, Q40, Q41, Q42, Q43, Q44, Q45	Congenital anomalies	Finland
Congenital anomalies of genital organs	752	752	Q50, Q51, Q52, Q53, Q54, Q55, Q56	Congenital anomalies	Finland
Congenital anomalies of urinary system	753	753	Q60, Q61, Q62, Q63, Q64	Congenital anomalies	Finland
Congenital anomalies of musculoskeletal system	754, 755, 756	754, 755, 756	Q65, Q66, Q67, Q68, Q69, Q70, Q 71, Q72, Q73, Q74, Q75, Q76, Q77, Q78, Q79	Congenital anomalies	Finland
Childhood allergy (age < 16)	--	6918, 477	J450, L20, J301, J302, J303, J304, L236, J3019	Other	Finland
Retention of urine	7861	7882	R33	Other	Finland
Fracture of femur	820	820	S72	Other	Finland
Toxic effect of ethanol	9800	9800	T510	Other	Finland

4.2. Diseases not included due to virtually complete lack of children

We excluded 11 rare diseases from the analysis for both women and men, since receiving diagnosis of these diseases resulted in an almost complete lack of children in the observational period (Supplementary **Table 6)**. Specifically, we filtered out diseases that had more than 30 individuals affected from the sibling-based design but less than 5 of affected individuals had live-born children at the end of their reproductive lifespan (up to 45 years old for women, 50 for men).

Supplementary **Table 6**: Diseases not included in the analysis due to resulting in an almost complete lack of children.

Disease	ICD-8	ICD-9	ICD-10	Category	Population
Lymphoid leukaemia	204	204	C91	Neoplasms	Finland, Sweden
Myeloid leukaemia	205	205	C92	Neoplasms	Finland, Sweden
Moderate intellectual disability	312	3180	F71	Mental-behavioural	Finland
Moderate intellectual disability	312	318A	F71	Mental-behavioural	Sweden
Severe intellectual disability	313	3181	F72	Mental-behavioural	Finland
Severe intellectual disability	313	318B	F72	Mental-behavioural	Sweden
Profound intellectual disability	314	3182	F73	Mental-behavioural	Finland
Profound intellectual disability	314	318C	F73	Mental-behavioural	Sweden
Other and unspecified intellectual disability	315	3199	F78, F79	Mental-behavioural	Finland
Other and unspecified intellectual disability	315	319	F78, F79	Mental-behavioural	Sweden
Muscular dystrophy	33030,33090	3591	G710	Nervous system	Finland
Muscular dystrophy	33030,33090	359B	G710	Nervous system	Sweden
Hemiplegia	34400	342	G81	Nervous system	Finland, Sweden
Other and unspecified paralytic syndromes	34408,34409	344	G838, G839	Nervous system	Finland, Sweden
Disorders of brain, other and unspecified	34798,34799	3488,3489	G938, G939, G948	Nervous system	Finland
Disorders of brain, other and unspecified	34798,34799	348W,348X	G938, G939, G948	Nervous system	Sweden
Congenital hydrocephalus	742	7423	Q03	Congenital anomalies	Finland

4.3. Disease prevalence from Finland and Sweden

Supplementary **Fig. 3**: Prevalence by age 45 in women (panel **a**) and 50 in men (panel **b**) for 213 disease diagnoses that were available in both Finland and Sweden. Each dot represents a disease diagnosis. Colors are assigned by disease categories.

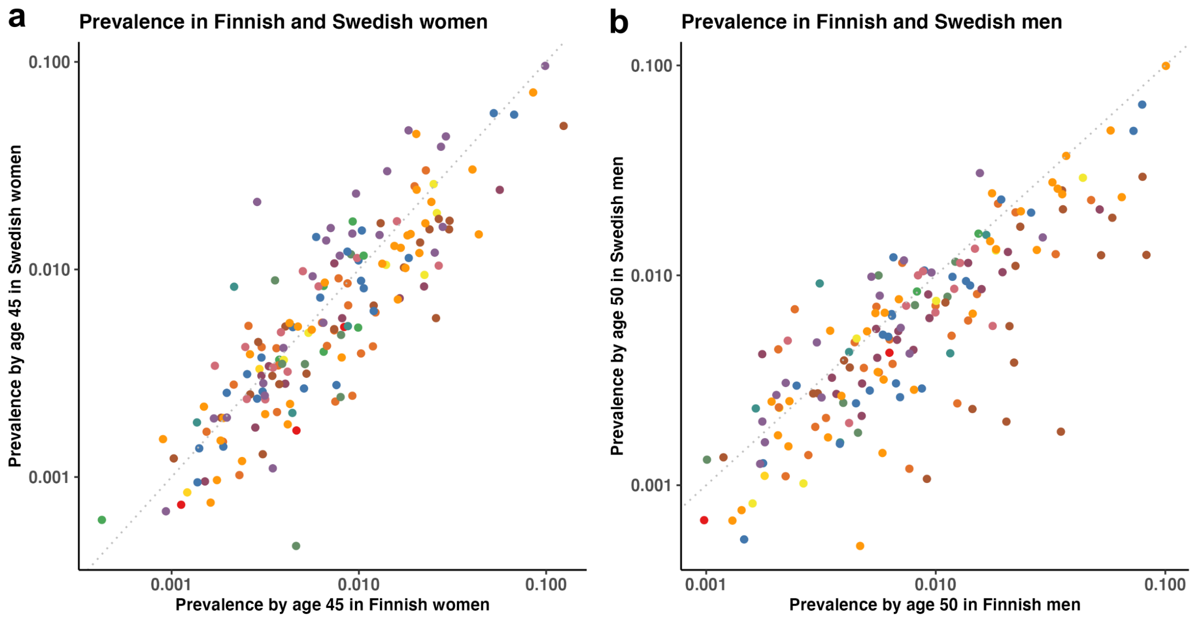

5. Relationship of disease diagnoses with childlessness

5.1. Effects of each disease diagnosis

We examined the relationship of 403 disease diagnoses with childlessness by age 45 in women and 50 in men in 71,524 pairs of full-sisters (35,544 in Finland and 35,980 in Sweden) and 77,622 full-brothers (38,928 in Finland and 38,694 in Sweden) who were discordant on childlessness, using a matched case-control design. Only sibling pairs who were also discordant on the disease status were used for the estimation of the disease effect. We therefore reported the number of sibling pairs discordant on both outcome and exposure for each country (Supplementary **Table 7**). Bonferroni correction was used to control for the familywise error rate.

For each disease diagnosis, we used a fixed-effect inverse-variance weighting method to aggregate summary statistics from two countries and tested for heterogeneity. Overall, the effects were highly consistent between Finnish and Swedish data and, after multiple testing corrections, only two diseases (acute alcohol intoxication and epilepsy) showed significantly heterogeneous effects between the two countries.

There were 74 disease diagnoses that were significantly associated with childlessness ($P<1.5\times10^{-4}$, after multiple-testing correction) in at least one sex, with 33 disease diagnoses shared among women and men. Results can also be explored on the interactive online dashboard available at https://dsgelrs.shinyapps.io/DiseaseSpecificLRS/.

Supplementary **Table 7**: Relationship of disease diagnoses with childlessness by age 45 in women and 50 in men, with a two-sided test.

(Supplementary **Table 7** is too large therefore see sheet "S7_EffectOnChildlessness" from "Supplementary_Table7_9_11_12_DiseaseSpecificChildlessness.xlsx"))

5.2. Effects by disease category

To quantify the average disease effects on childlessness for each disease category, we meta-analyzed effects for diseases of the same category for each sex using a random-effect model to account for heterogeneity across different diseases. Among all 16 disease categories, on average, mental-behavioral, congenital anomalies, and endocrine-nutritional-metabolic disorders had the strongest association (Supplementary **Table 8**).

Supplementary **Table 8**: Relationship of disease diagnoses with childlessness by age 45 in women and 50 in men by disease category, with a two-sided test.

Category	Sex	OR	OR [95% CI lower]	OR [95% CI upper]	P value	P value from heterogeneity test
Infectious-parasitic	Women	1.07	0.97	1.18	0.20	1.62E-03
Infectious-parasitic	Men	0.97	0.90	1.05	0.51	0.43
Neoplasms	Women	1.16	0.77	1.75	0.47	7.14E-09
Neoplasms	Men	1.21	0.79	1.83	0.38	2.03E-07
Blood and immune mechanism	Women	1.24	0.95	1.61	0.11	0.18
Blood and immune mechanism	Men	1.09	0.83	1.42	0.54	0.7
Endocrine-nutritional-metabolic	Women	1.39	1.06	1.84	0.02	1.35E-10
Endocrine-nutritional-metabolic	Men	2.02	1.59	2.57	7.37E-09	0.08
Mental-behavioural	Women	3.08	2.55	3.72	1.25E-31	4.50E-71
Mental-behavioural	Men	3.22	2.61	3.97	6.79E-28	7.86E-128
Nervous system	Women	1.82	1.08	3.08	0.03	1.82E-43
Nervous system	Men	1.62	1.03	2.56	0.04	2.67E-45
Eye and adnexa	Women	1.31	1.12	1.52	7.01E-04	0.01
Eye and adnexa	Men	1.53	1.24	1.89	6.16E-05	7.75E-14
Ear and mastoid process	Women	1.37	1.16	1.61	1.43E-04	0.12
Ear and mastoid process	Men	1.13	0.96	1.34	0.13	0.01
Circulatory system	Women	1.70	1.32	2.19	3.35E-05	1.17E-16
Circulatory system	Men	1.37	1.11	1.69	3.36E-03	3.34E-16
Respiratory system	Women	1.12	1.02	1.24	0.02	2.06E-23
Respiratory system	Men	0.97	0.91	1.02	0.23	1.71E-11
Digestive system	Women	1.10	1.00	1.21	0.05	8.33E-08
Digestive system	Men	1.26	1.13	1.40	1.91E-05	3.53E-19
Skin and subcutaneous tissue	Women	1.01	0.90	1.13	0.84	0.02
Skin and subcutaneous tissue	Men	1.04	0.98	1.10	0.23	0.66
Musculoskeletal system	Women	1.12	1.01	1.25	0.03	1.86E-16
Musculoskeletal system	Men	0.88	0.82	0.94	2.67E-04	2.88E-08
Genitourinary system	Women	0.97	0.88	1.07	0.52	1.58E-11
Genitourinary system	Men	1.10	1.00	1.21	0.05	2.86E-03
Congenital anomalies	Women	3.55	2.57	4.89	1.15E-14	0.01
Congenital anomalies	Men	1.83	1.34	2.50	1.62E-04	0.01
Other	Women	1.25	0.85	1.84	0.25	0.02
Other	Men	1.10	0.91	1.33	0.32	0.21

5.3. Age-of-onset-dependent effects

We hypothesized that the age when the disease was first diagnosed, a proxy for the age of onset, would influence the chances of being childless by either capturing disease severity or directly impacting factors underlying individuals' reproductive trajectory. Therefore, for disease diagnoses that were significantly associated with childlessness, we assessed whether there were any age-of-onset-dependent effects by considering the age of onset (8 groups <=15, 16-20, 21-25, 26-30, 31-35, 36-40, 41-45, or unaffected for both sexes and an additional group (46-50) for men) as fixed effects in conditional logistic regression. The analysis was performed separately for each disease (Supplementary **Table 9**) in 71,524 full-sister and 77,622 full-brother pairs who were discordant on childlessness. We then meta-analyzed disease effects for each age of onset group using a random-effect model (Supplementary **Table 10**).

Supplementary **Table 9**: Age-of-onset-dependent effects for each disease diagnosis that significantly increased childlessness, with a two-sided test.

(Supplementary **Table 9** is too large therefore see sheet "S9_AgeOnsetEffects" from "Supplementary_Table7_9_11_12_DiseaseSpecificChildlessness.xlsx")

Supplementary **Table 10**: Age-of-onset-dependent effects across all disease diagnoses that significantly increased childlessness, with a two-sided test.

**Sex**	**Age onset group**	**OR**	**OR [95% CI lower]**	**OR [95% CI upper]**	**P value**	**P value from heterogeneity test**
Women	<=15	1.89	1.55	2.31	5.59E-10	1.24E-15
Women	16-20	2.53	1.98	3.25	2.05E-13	2.78E-21
Women	21-25	3.12	2.53	3.84	1.02E-26	1.88E-15
Women	26-30	2.68	2.12	3.38	1.18E-16	1.38E-08
Women	31-35	1.74	1.33	2.29	6.35E-05	3.08E-04
Women	36-40	1.24	0.55	2.80	0.60	0.02
Men	<=15	2.27	1.86	2.77	5.89E-16	2.18E-05
Men	16-20	2.38	1.82	3.12	2.61E-10	4.24E-19
Men	21-25	2.81	2.20	3.60	2.38E-16	9.81E-50
Men	26-30	3.07	2.45	3.85	4.07E-22	1.34E-09
Men	31-35	2.57	1.99	3.32	5.82E-13	2.64E-03
Men	36-40	1.76	1.13	2.75	0.01	0.06
Men	41-45	1.03	0.49	2.17	0.93	0.35

5.4. Stratification by parity for individuals with children

We also know that fertility transitions are conditioned on previous births and for that reason a parity-specific analysis is essential[46]. We therefore also look beyond the average fertility level of a country, since the average obscures the distribution of parity and polarization within particular subgroups. This is particularly important when examining childlessness in parity polarised countries[47].

For individuals with children, we assessed whether the effects of diseases were consistent across parities by comparing childless individulas to those with one child, two children, or more than two children. When comparing childless individuals to their siblings with one child, nine out of 28 disease diagnoses examined in women and 15 out of 29 disease diagnoses in men remained significantly associated with childlessness (Supplementary **Table 11**). Compared to main analyses (childless individuals versus those with at least one child), 14 disease diagnoses in women and six in men had significantly reduced ORs (Supplementary **Fig. 4a-b**). For example, in women, the OR of schizophrenia on childlessness dropped from 11.6 [9.1-14.9] to 5.1 [3.7-7.0] (P difference=6.9×10^{-5}) when restricting to individuals with one child. For individuals with high parities, such as having exactly two children (Supplementary **Fig. 4c-d**) or at least three or more children (Supplementary **Fig. 4e-f**), limited differences in ORs were observed compared to the main analyses. Jointly, these results indicated that, in Nordic countries such as Finland and Sweden, individuals with one child were more impacted by disease status than individuals with high parities, but to a less degree than individuals with no biological children.

Supplementary **Table 11**. Relationship of disease diagnoses with childlessness by age 45 in women and 50 in men stratified by parity of individuals with children, with a two-sided test.

(Supplementary **Table 11** is too large therefore see sheet "S11_StrataParity" from "Supplementary_Table7_9_11_12_DiseaseSpecificChildlessness.xlsx")

Supplementary **Fig. 4**: Relationship of disease diagnoses with childlessness by age 45 in women (panel a, c, and e) and 50 in men (panel b, d, and f) from main analysis (childless individuals were compared to those with at least one child) versus the analysis stratified by parities (childless individuals were compared to those with one child (panel a and b), with two children (panel c and d), or with at least three or more children (panel e and f)), for disease diagnoses that were significantly increased childlessness from the main analysis.

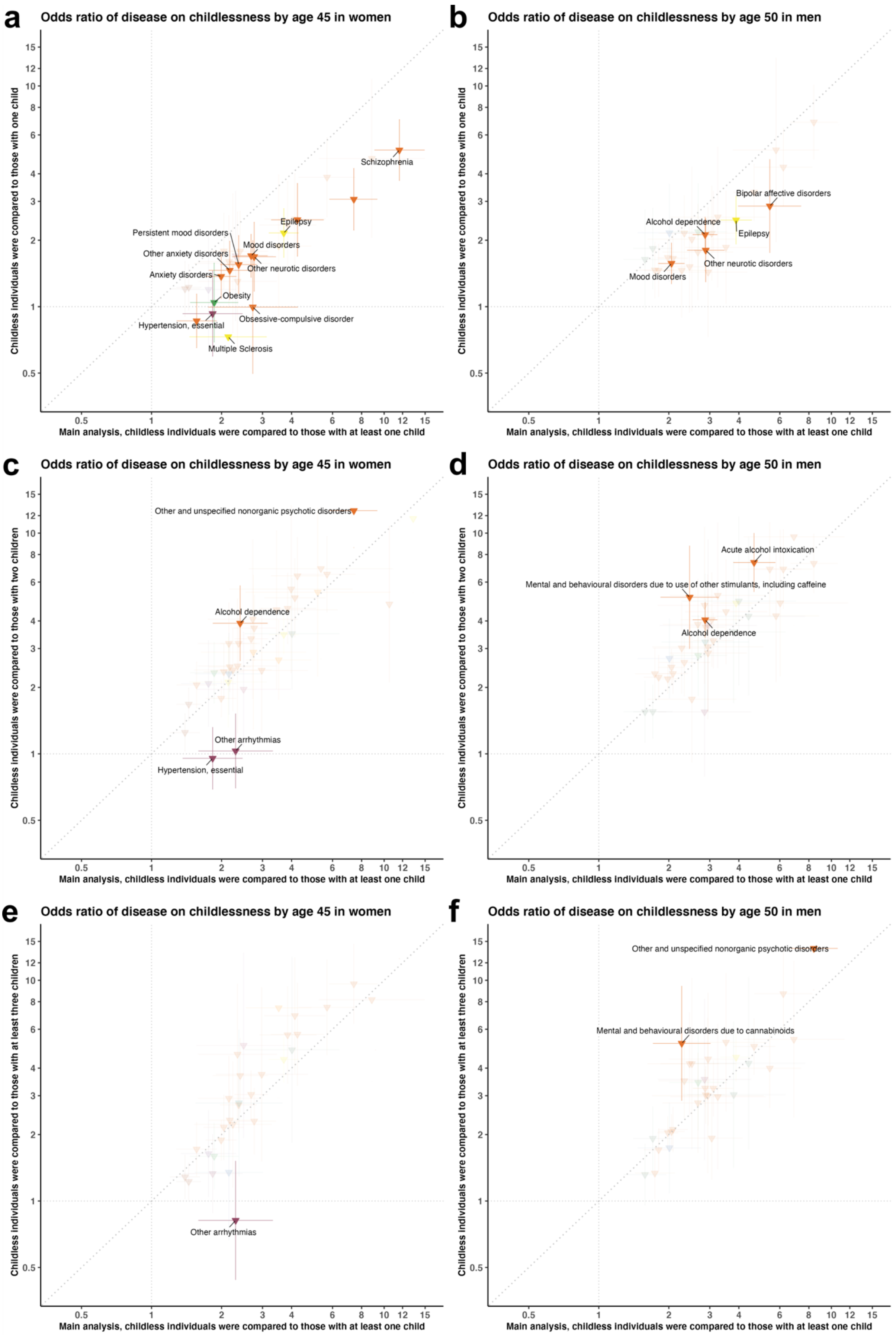
a
Odds ratio of disease on childlessness by age 45 in women
Childless individuals were compared to those with one child
Main analysis, childless individuals were compared to those with at least one child
Schizophrenia
Epilepsy
Persistent mood disorders
Mood disorders
Other anxiety disorders
Other neurotic disorders
Anxiety disorders
Obesity
Obsessive-compulsive disorder
Hypertension, essential
Multiple Sclerosis
b
Odds ratio of disease on childlessness by age 50 in men
Bipolar affective disorders
Alcohol dependence
Epilepsy
Other neurotic disorders
Mood disorders
c
Odds ratio of disease on childlessness by age 45 in women
Childless individuals were compared to those with two children
Other and unspecified nonorganic psychotic disorders
Alcohol dependence
Other arrhythmias
Hypertension, essential
d
Odds ratio of disease on childlessness by age 50 in men
Acute alcohol intoxication
Mental and behavioural disorders due to use of other stimulants, including caffeine
Alcohol dependence
e
Odds ratio of disease on childlessness by age 45 in women
Childless individuals were compared to those with at least three children
Other arrhythmias
f
Odds ratio of disease on childlessness by age 50 in men
Other and unspecified nonorganic psychotic disorders
Mental and behavioural disorders due to cannabinoids
0.5
1
2
3
4
6
8
10
12
15

6. Mediation effect by singlehood

Similar to our previous childlessness analysis, we estimated the effect of 403 disease diagnoses on the risk of being without a partner by age 45 in women and 50 in men. For index individuals in Finland, we obtained their longitudinal marriage and partnership information between 11 April 1971 and 31 December 2018 from the Population Information System. In Sweden, we collected information of married couples and cohabiting unions with biological children between 1 January 1977 and 31 December 2017 from Statistics Sweden. We defined individuals as partnerless if they did not have any abovementioned marriage or partnership registered by age 45 (women) or 50 (men).

6.1. Effects on singlehood

Supplementary **Fig. 5**: Relationship of 402 disease diagnoses with being without a partner by age 45 in women (panel **a**) and 50 in men (panel **b**). Only disease diagnoses that are significantly associated after multiple-testing correction are colored.

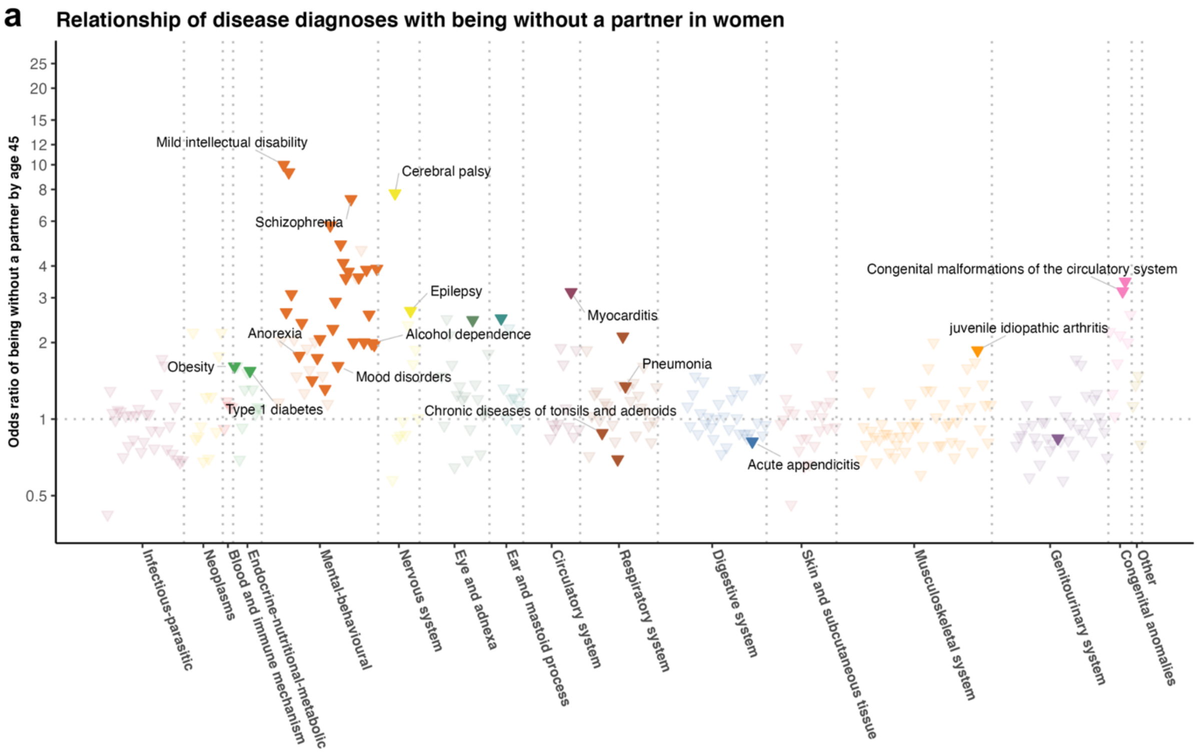

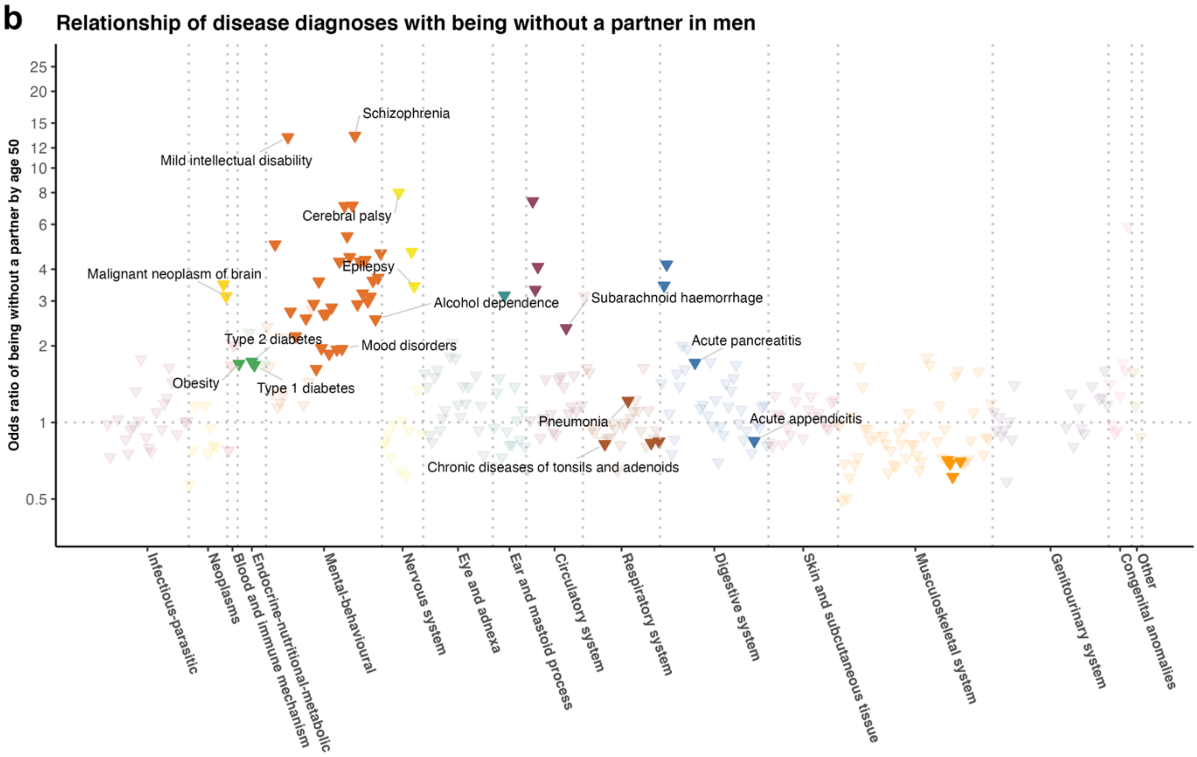

Supplementary **Fig. 6**: Sex-specific effects for the association between disease diagnoses and being without a partner by age 45 in women and 50 in men, for 68 disease diagnoses that were significantly associated with increased childlessness from the main analysis. Only disease diagnoses that are significantly different between sexes at a nominal P value (no adjustments were made for multiple comparisons) with a two-sided t-test are colored.

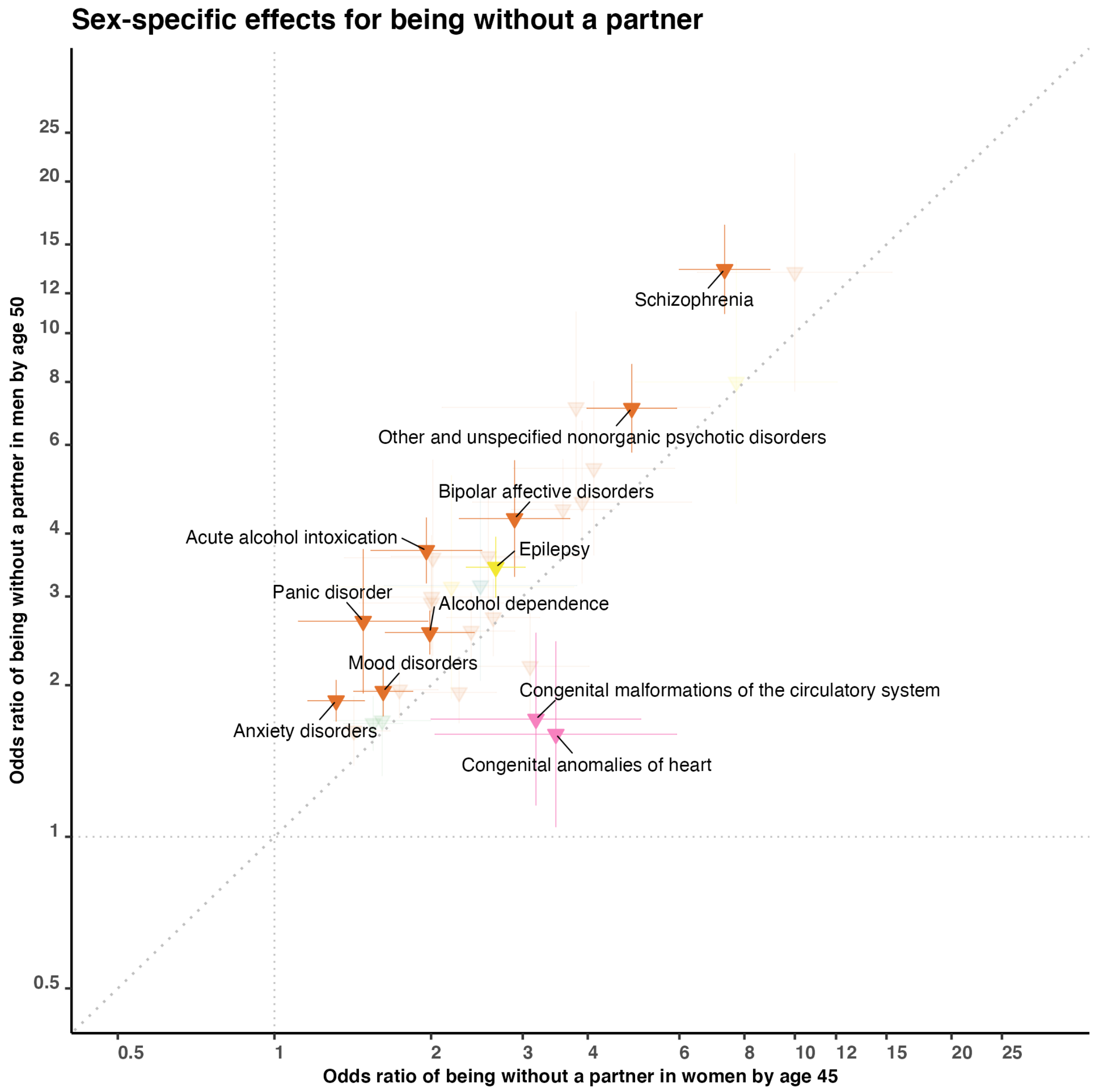

6.2. Mediation analysis

To quantify the overall effect of singlehood on the association between disease diagnoses and childlessness, we performed linear regression for each sex using all disease diagnoses that were significantly associated with childlessness, with log ORs of disease diagnoses on childlessness as the dependent variable and log ORs on singlehood as the independent variable (Supplementary **Fig. 7**). High R^2 was observed in both men (R^2=0.85, regression coefficient =1.08, P=3.5×10^{-23}) and women (R^2=0.71, regression coefficient =0.96, P=1.0×10^{-15}). Also, the intercept was significant in women (0.42, P=1.0×10^{-15}) but not in men (0.11, P=0.13), demonstrating that there were additional effects not fully captured by union formation in women.

Supplementary **Fig. 7**: Relationship of disease diagnoses with childlessness (main analysis) versus singlehood by age 45 in women (panel **a**) and 50 in men (panel **b**), for 74 disease diagnoses that were significantly associated with childlessness from the main analysis. Only disease diagnoses that are significantly different between two outcomes at a nominal P value (no adjustments were made for multiple comparisons) with a two-sided t-test are colored.

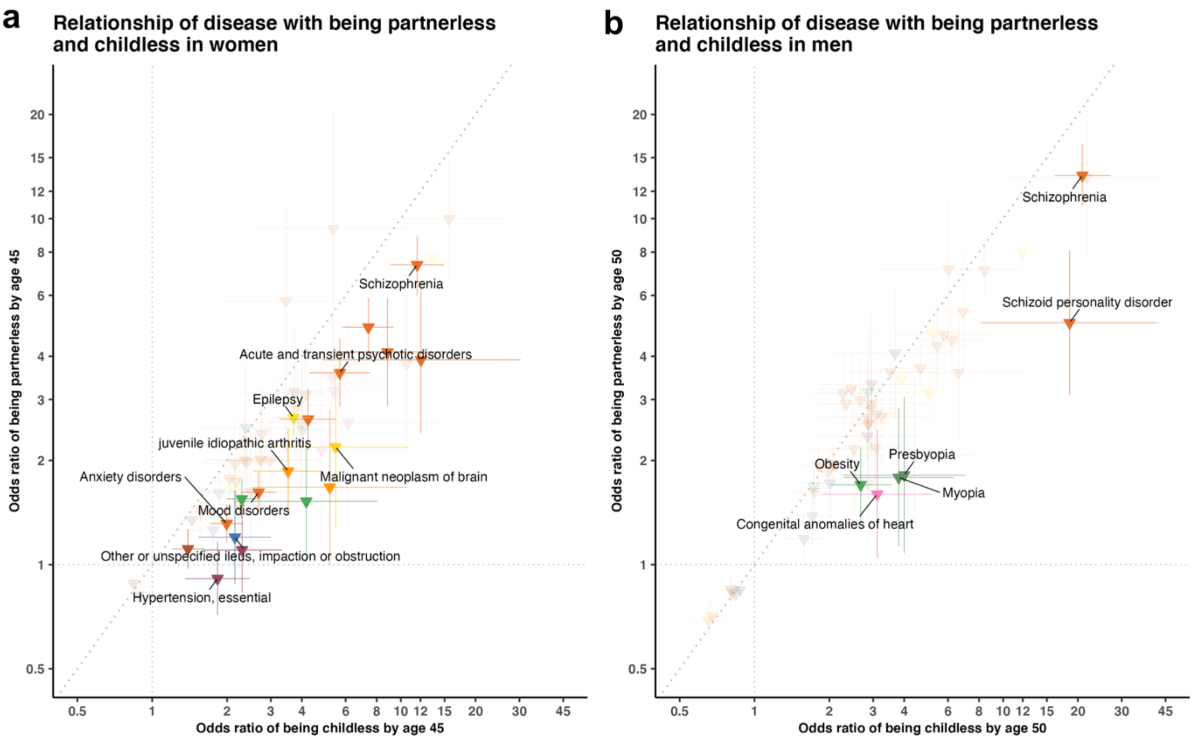

For each disease diagnosis, we performed a causal mediation analysis, with singlehood as a dichotomous latent factor. A median of 29.3% of the disease effects on childlessness in women and 37.9% in men was mediated by singlehood (Supplementary **Table 12**). Different patterns were observed across diseases. For example, singlehood was a significant mediator for women diagnosed with schizophrenia (OR=2.1 [2.0-2.2] for indirect and 6.8 [5.0-9.2] for direct effect), but not for women with hypertension (1.2 [1.1-1.4] for indirect and 2.4 [1.6-3.5] for direct effect).

Supplementary **Table 12**: Direct and indirect effects (mediated by singlehood) of disease on childlessness estimated from mediation analysis, with a two-sided test.

(Supplementary **Table 12** is too large therefore see sheet “S12_MediationAnalysis” from “Supplementary_Table7_9_11_12_DiseaseSpecificChildlessness.xlsx”)

6.3. Effects among individuals with registered partners

Supplementary **Fig. 8**: Relationship of 169 disease diagnoses with childlessness among women (by age 45) (panel **a**) and men (by age 50) (panel **b**) with registered partners. Only disease diagnoses that are significantly associated after multiple-testing correction are colored.

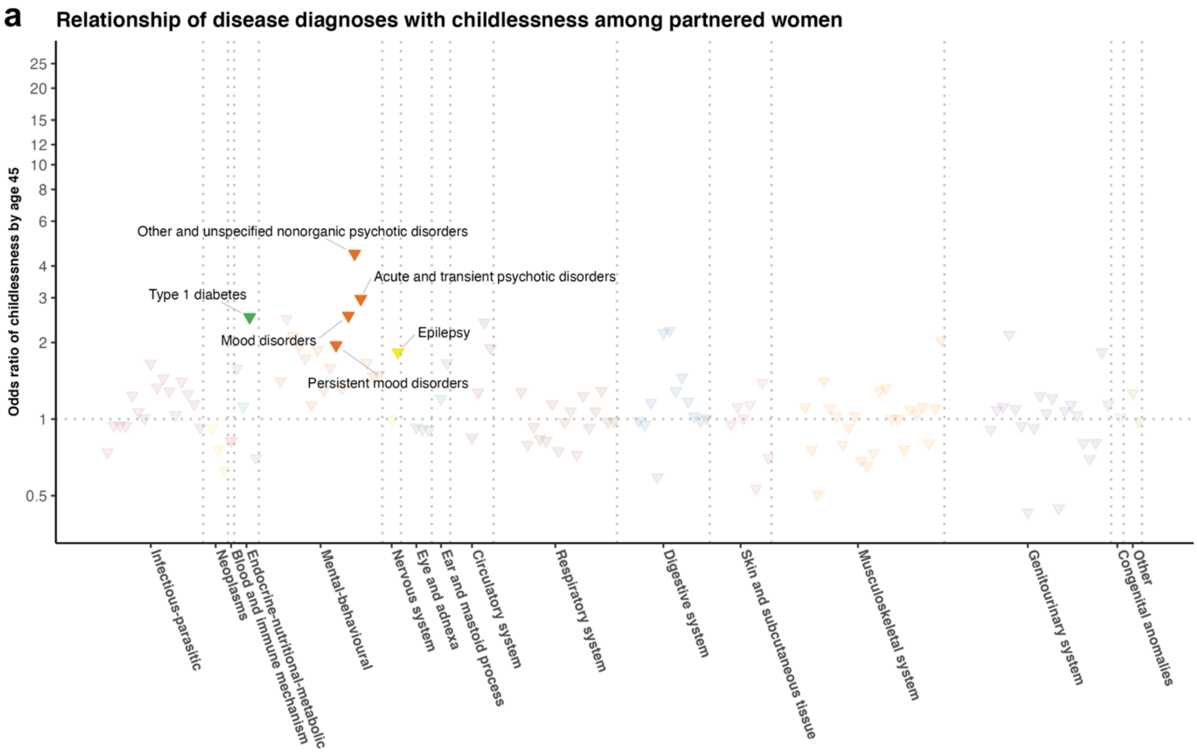

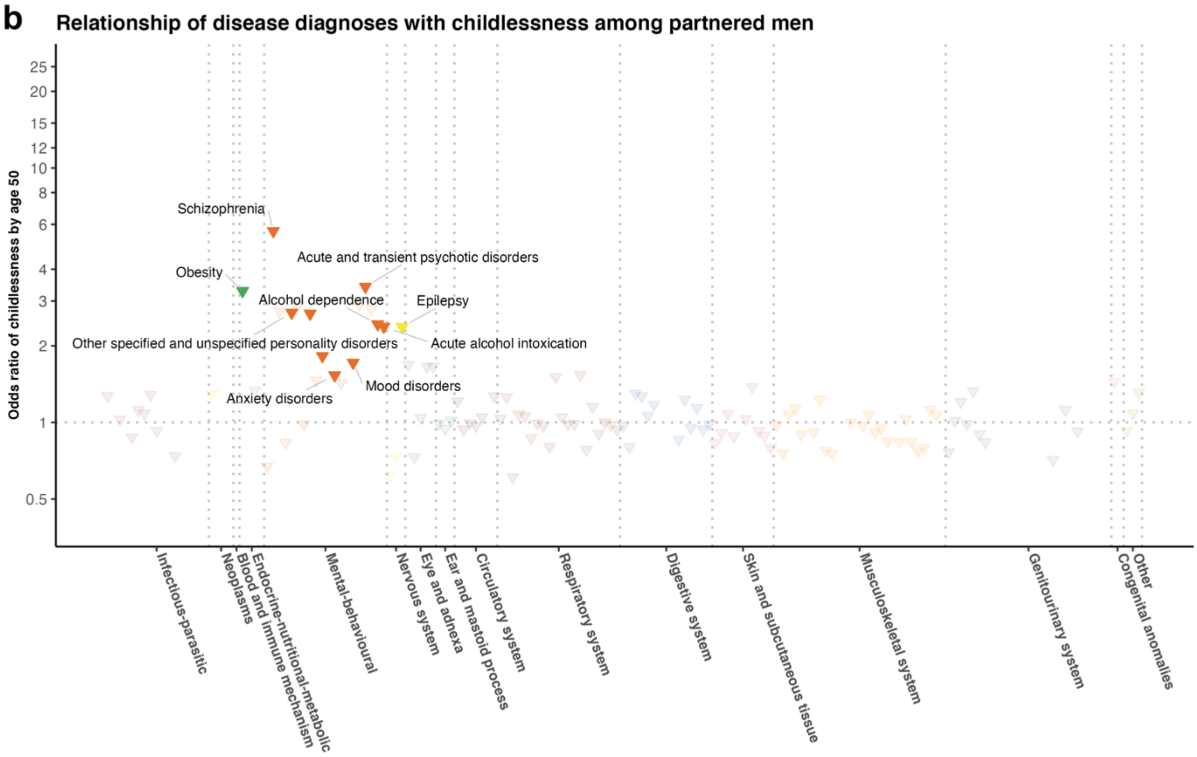

Supplementary **Fig. 9**: Relationship of disease diagnoses with childlessness by age 45 in women (panel **a**) and 50 in men (panel **b**) from the general population (main analysis) versus people with registered partners, for 36 disease diagnoses that were significantly associated with childlessness from the main analysis.

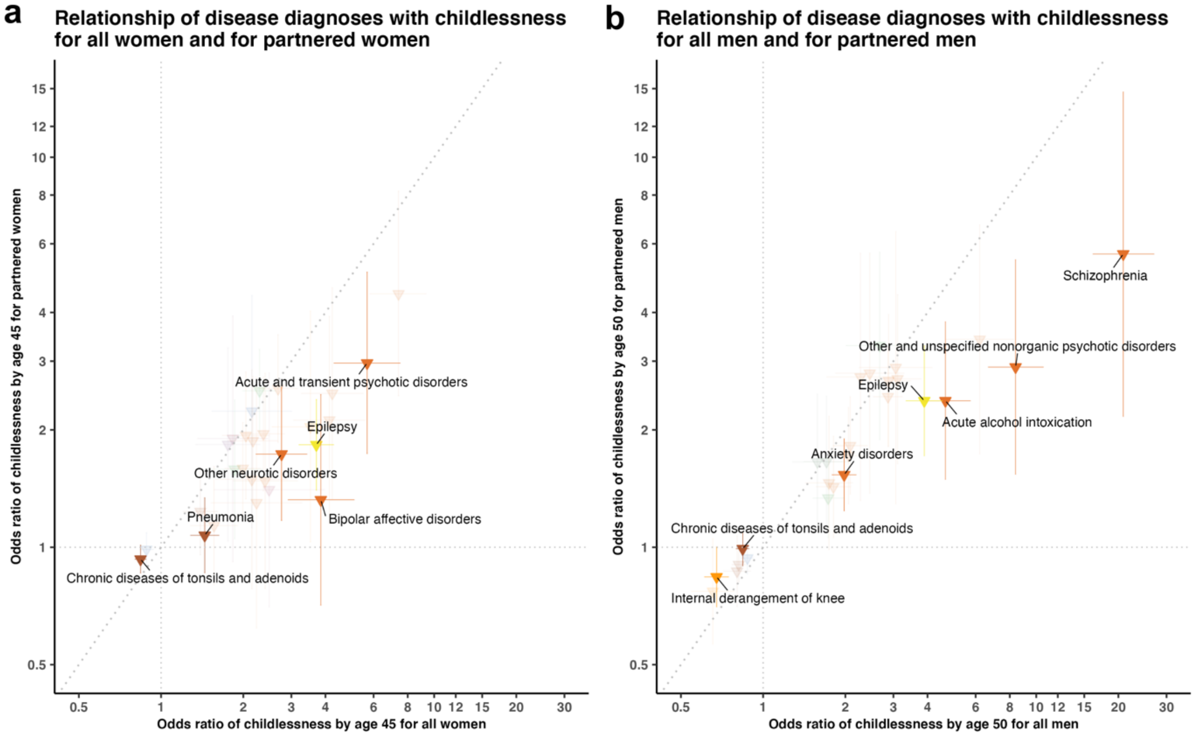

7. Population-based and sensitivity analyses

To estimate the population-level effect of diseases on childlessness we performed a nested incident-matched case-control design which matched each childless case to one control based on sex, birth year, municipality of birth (545 from Finland and 1,091 from Sweden), and the highest parental education level (International Standard Classification of Education 1997). For the matching process, among people with the same birth municipality and parental education background, we firstly searched for the individual born at the same year as the control and then extended to the individual with the closest birth year (up to 5 years of difference). There was no overlap among matched controls. Similar to the sibling-based analysis, we only considered the disease diagnosis onset at least one year before the age at first birth of the matched control and examined associations between diseases and childlessness using a conditional logistic regression model. In total, we used 226,860 matched pairs of women (107,375 in Finland and 119,485 in Sweden) and 274,941 of men (127,689 in Finland and 147,252 in Sweden) for the population-based analysis.

Supplementary **Fig. 10**: Relationship of 403 disease diagnoses with childlessness by age 45 in women (panel **a**) and 50 in men (panel **b**) from using a nested incident-matched case-control design, matched by birth year, birth municipality, and highest parental education level. Only disease diagnoses that are significantly associated after multiple-testing correction are colored.

a Relationship of disease diagnoses with childlessness in women from population-based analysis

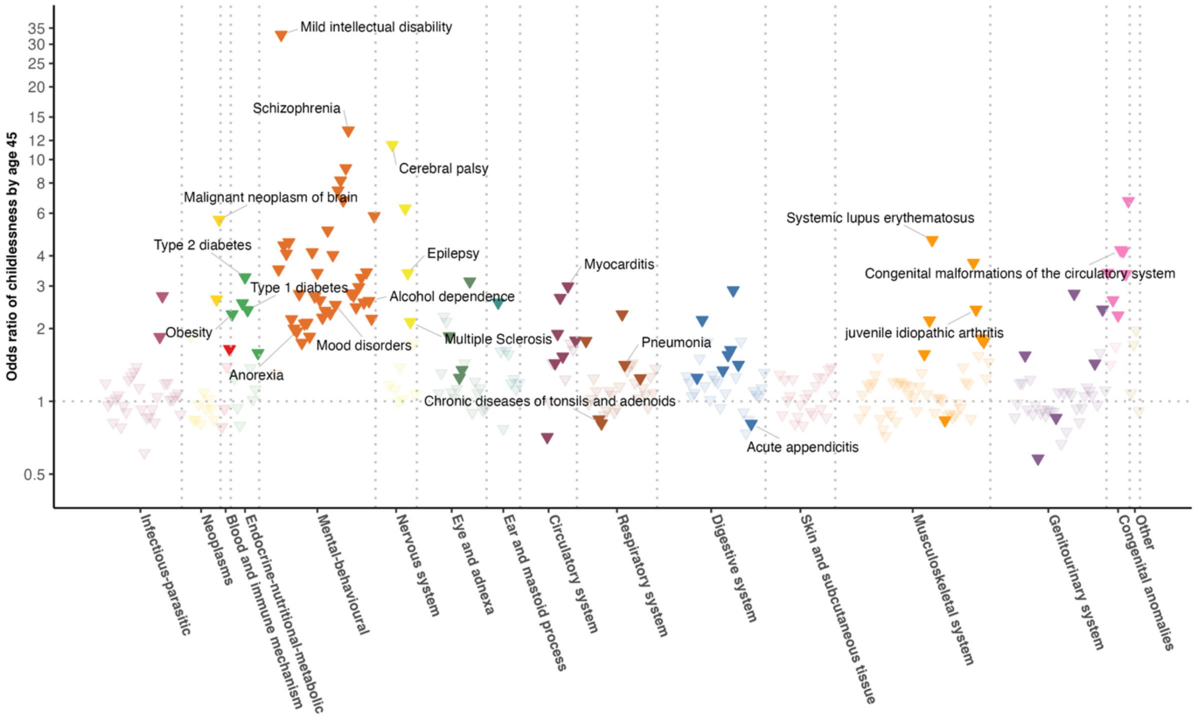

b Relationship of disease diagnoses with childlessness in men from population-based analysis

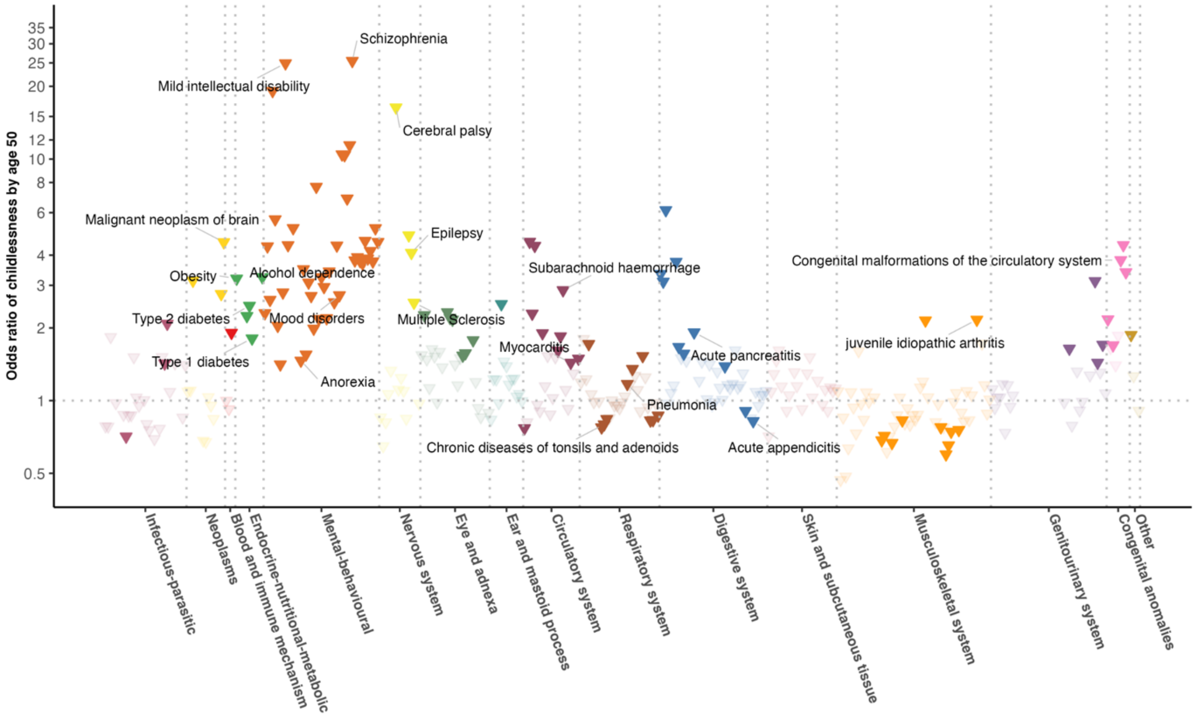

Supplementary **Fig. 11**: Relationship of disease diagnoses with childlessness by age 45 in women (panel **a**) and 50 in men (panel **b**) from the sibling-based (main analysis) versus population-based analyses, for 74 disease diagnoses that were significantly associated with childlessness from the main analysis.

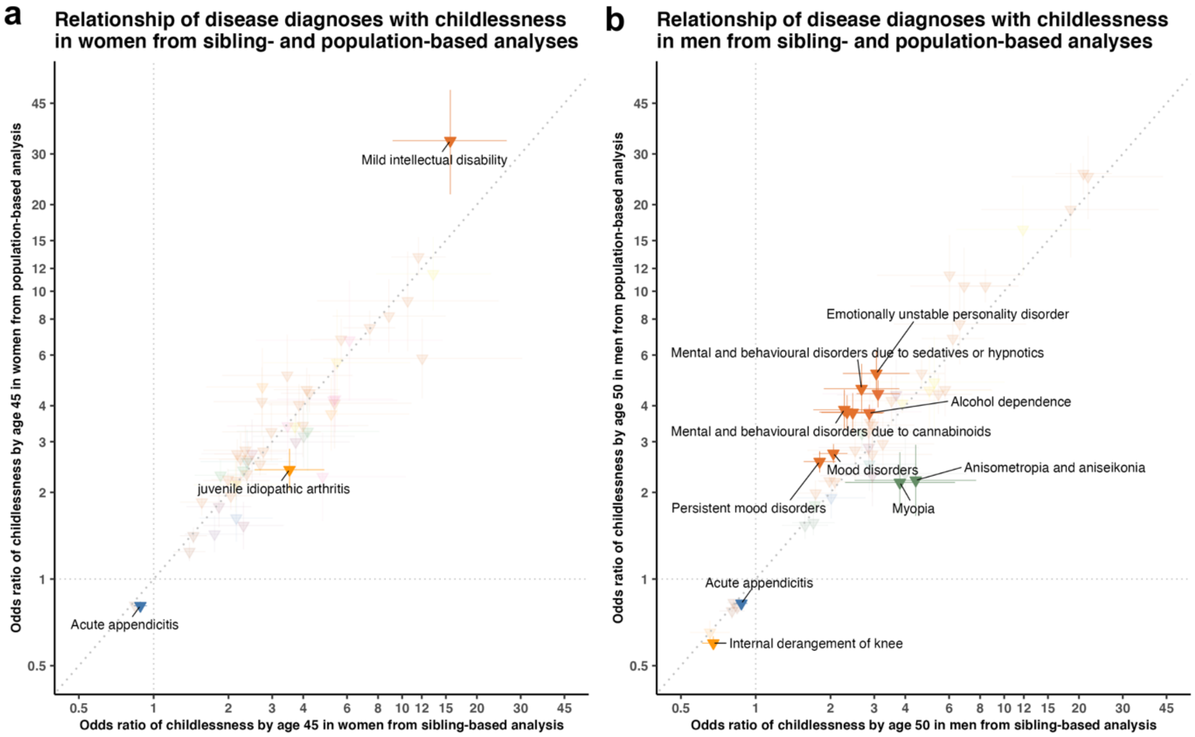

Supplementary **Fig. 12**: Relationship of 365 disease diagnoses with childlessness restricted to women alive by age 45 (panel **a**) and men by 50 (panel **b**), using a sibling-based design. Only disease diagnoses that are significantly associated after multiple-testing correction are colored.

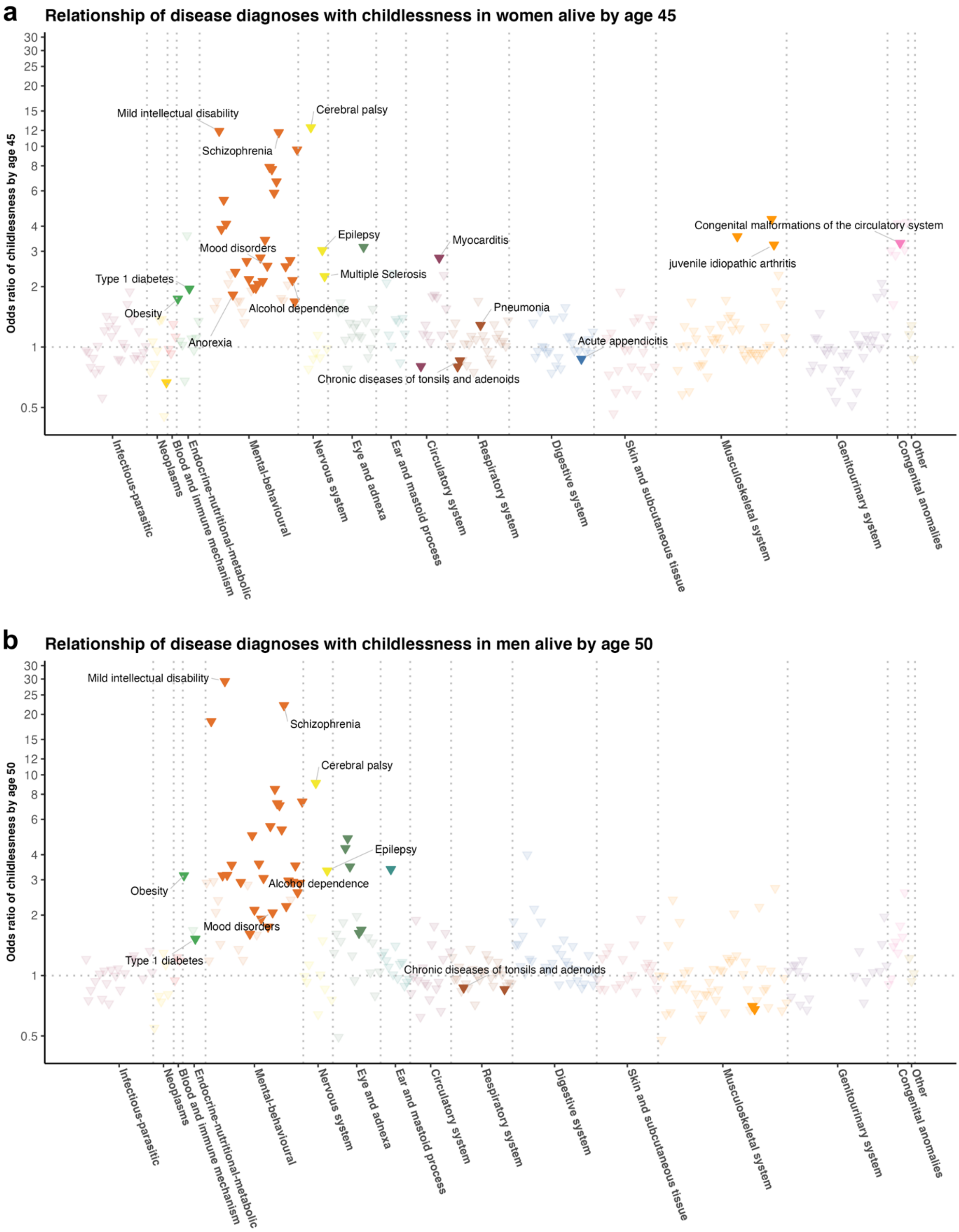

Supplementary **Fig. 13**: Relationship of disease diagnoses with childlessness in the general population (main analysis) versus the sensitivity analysis which restricted to women alive by age 45 (panel **a**) and men by 50 (panel **b**), for 71 disease diagnoses that were significantly associated with childlessness from the main analysis.

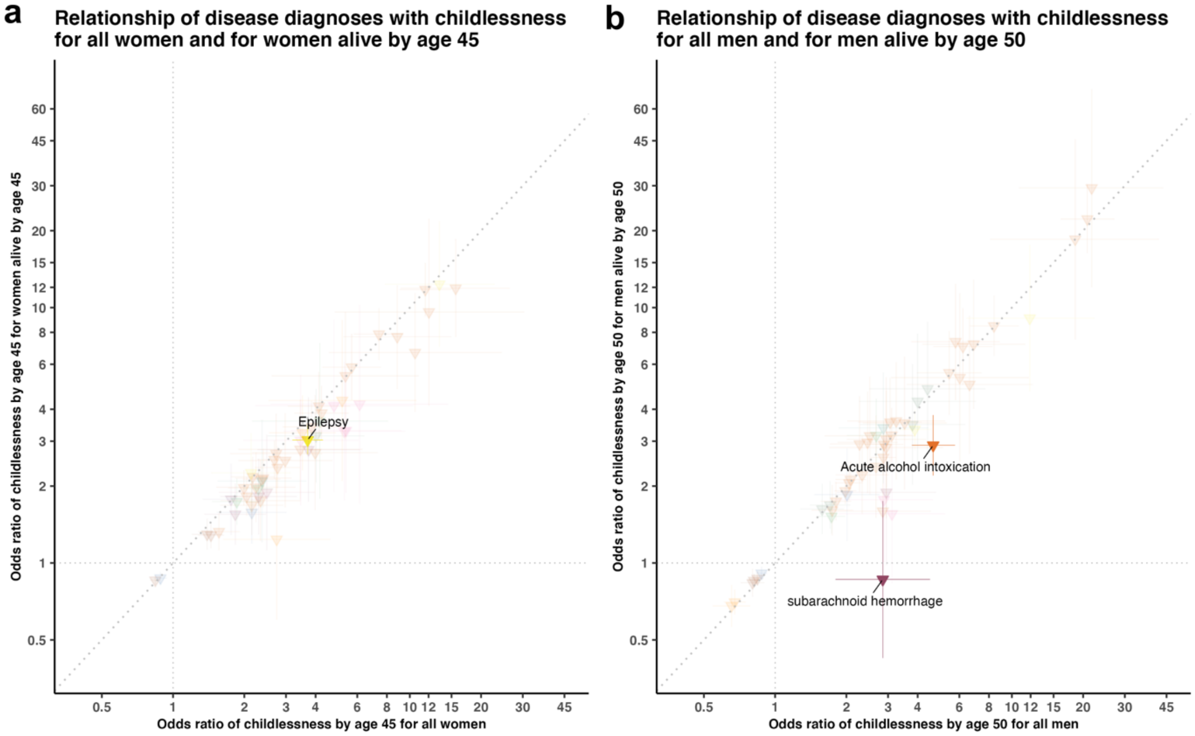

Supplementary **Fig. 14**: Relationship of 403 disease diagnoses with childlessness by age 45 in women (panel **a**) and 50 in men (panel **b**) from using a Cox proportional hazards regression model stratified by same-sex full-siblings. Only disease diagnoses that are significantly associated after multiple-testing correction are colored.

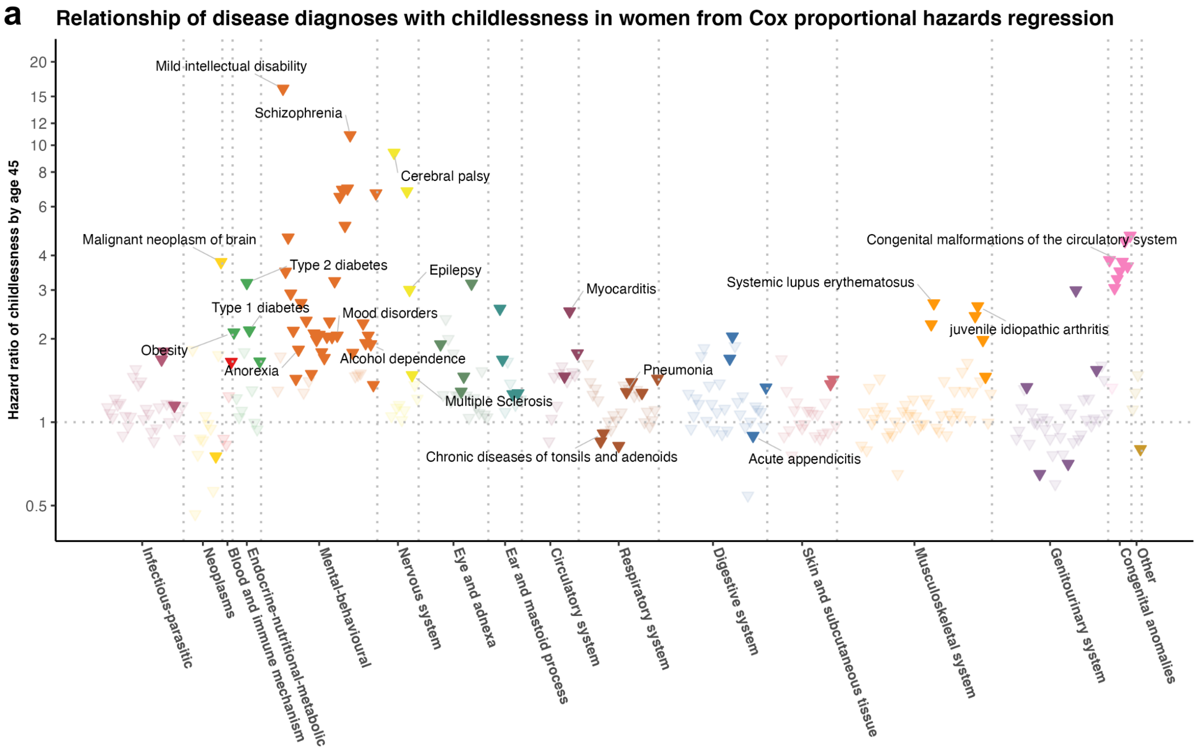

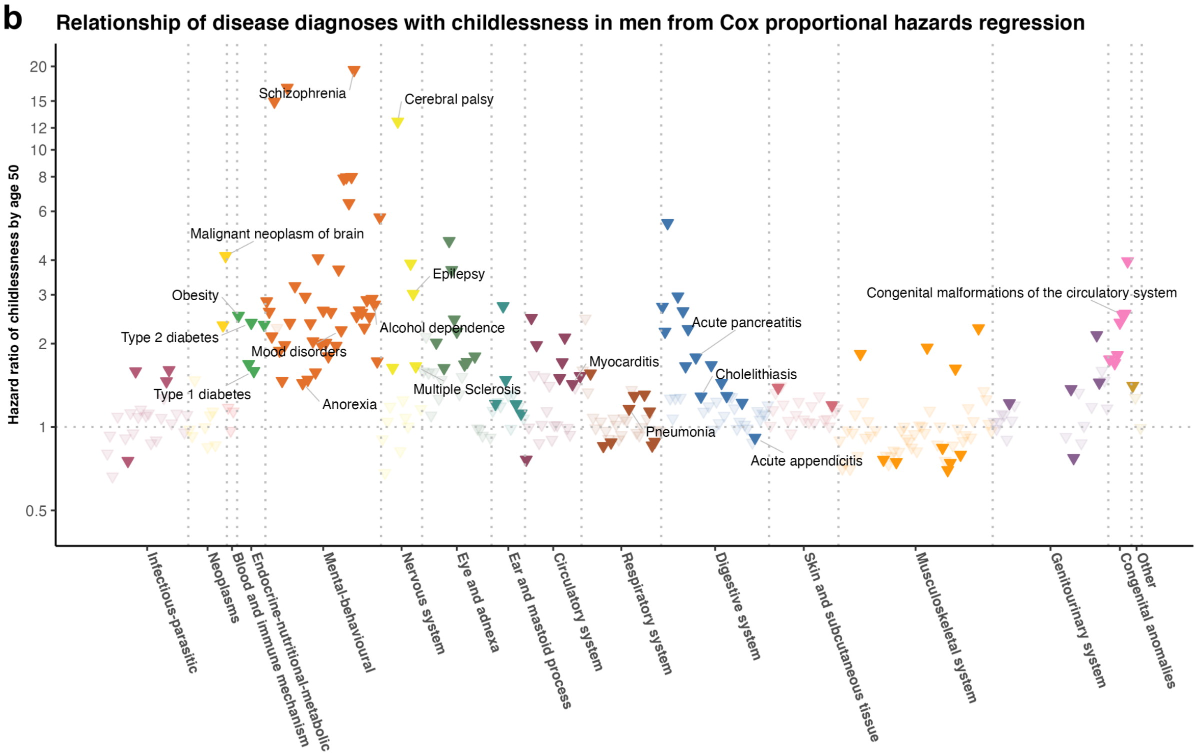

Supplementary **Fig. 15**: Relationship of disease diagnoses with childlessness by age 45 in women (panel **a**) and 50 in men (panel **b**) from conditional logistic regression (main analysis) versus stratified Cox proportional hazards regression, for 74 disease diagnoses that were significantly associated with childlessness from the main analysis.

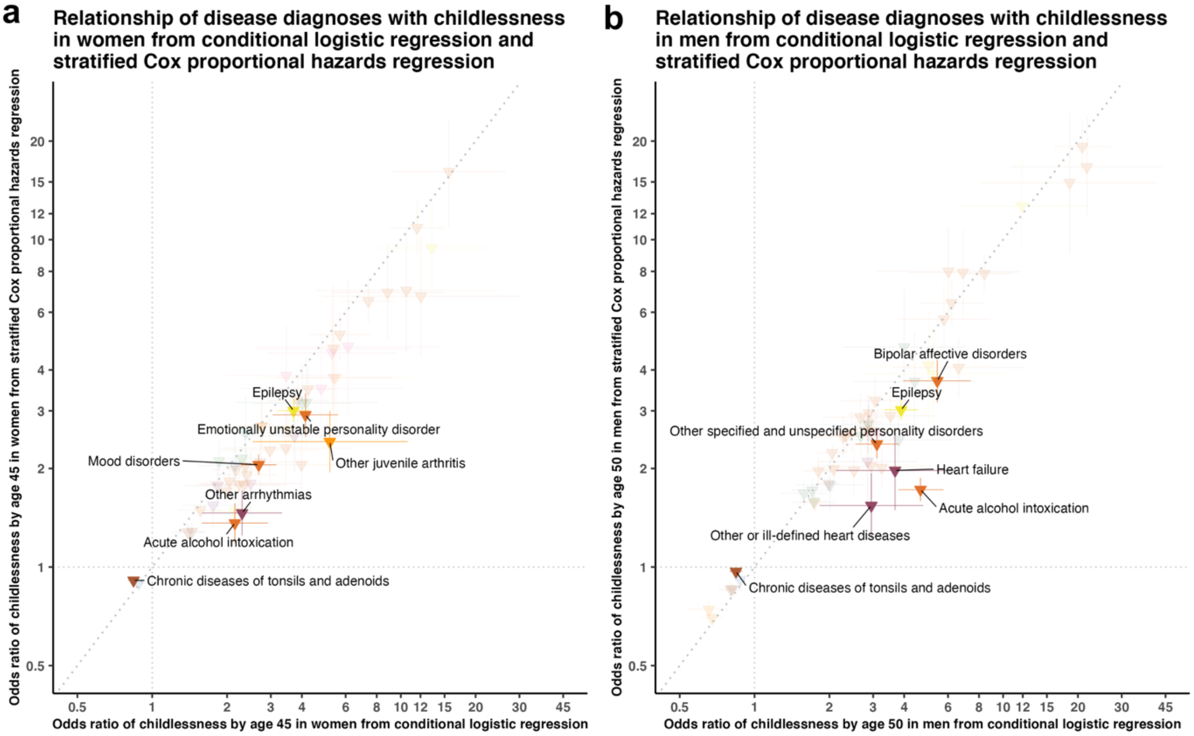

Supplementary **Fig. 16**: Relationship of disease diagnoses with childlessness by age 45 in women (panel **a**) and 50 in men (panel **b**) from main analysis versus sensitivity analysis adjusting for individuals’ highest education level, for 74 disease diagnoses that were significantly associated with childlessness from the main analysis.

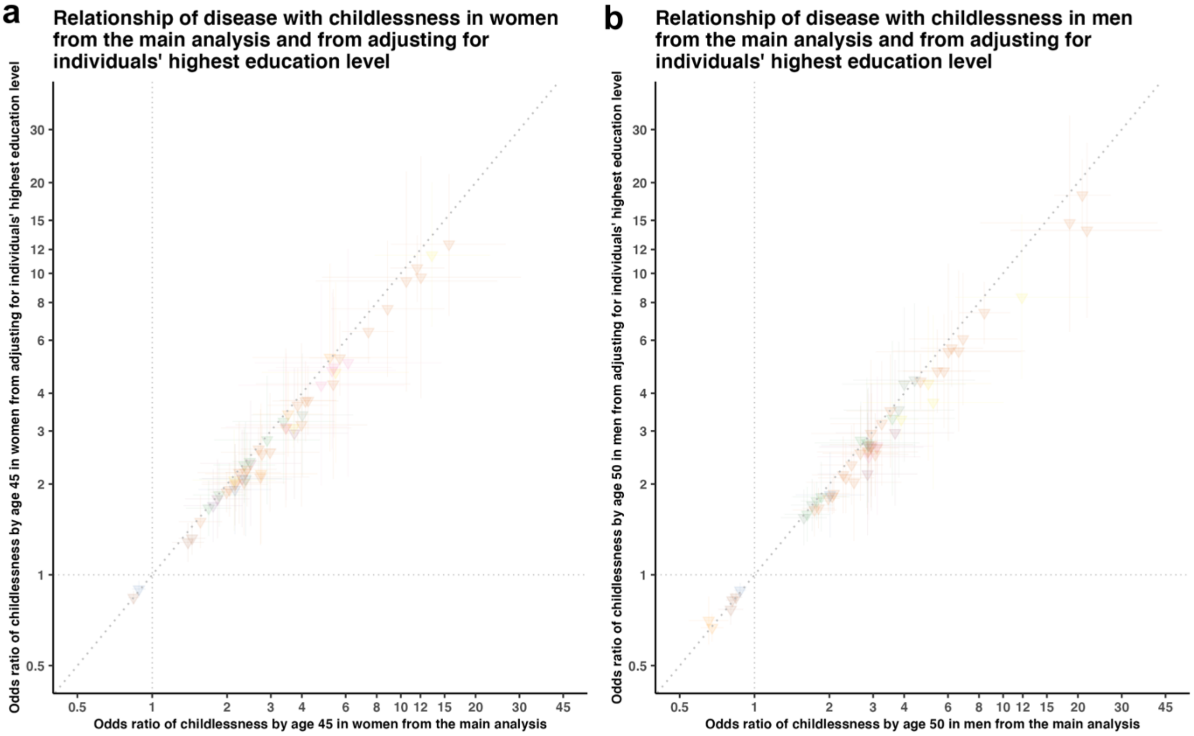

Supplementary **Fig. 17**: Relationship of disease diagnoses with childlessness by age 45 in women (panel **a**) and 50 in men (panel **b**) from population-based analyses with or without adjusting for individuals' highest education level, for 74 disease diagnoses that were significantly associated with childlessness from the main analysis.

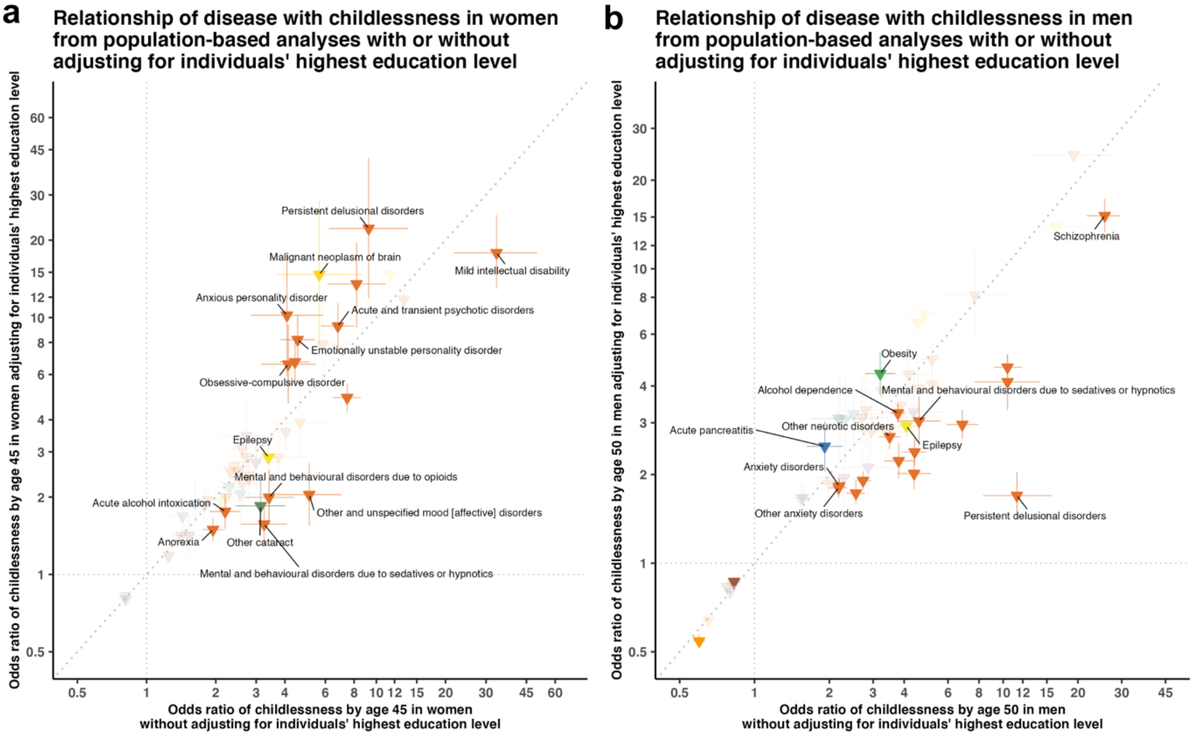

Reference

1. Kreyenfeld, M. and Konietzka, D., 2017. Analyzing childlessness (pp. 3-15). Springer International Publishing.
2. Tanturri, M.L. and Mencarini, L., 2008. Childless or childfree? Paths to voluntary childlessness in Italy. Population and development review, 34(1), pp.51-77.
3. Stephansson O, Dickman PW, Johansson AL, Cnattingius S. The influence of socioeconomic status on stillbirth risk in Sweden. Int J Epidemiol 2001; **30**: 1296-1301.
4. Briley DA, Tropf FC, Mills MC. What explains the heritability of completed fertility? Evidence from two large twin studies. Behav Genet 2017; 47: 36-51.
5. Alwan S, Yee IM, Dybalski M, et al. Reproductive decision making after the diagnosis of multiple sclerosis (MS). Mult Scler J 2013; 19: 351-358.
6. Currie J, Schwandt H. Short-and long-term effects of unemployment on fertility. PNAS 2014; 111: 14734-14739.
7. Collaborative Group on Hormonal Factors in Breast Cancer. Breast cancer and breastfeeding: collaborative reanalysis of individual data from 47 epidemiological studies in 30 countries, including 50 302 women with breast cancer and 96 973 women without the disease. Lancet 2002; 360: 187-195.
8. Mills MC, Rindfuss RR, McDonald P, Te Velde E. Why do people postpone parenthood? Reasons and social policy incentives. Hum Reprod Update 2011; 17: 848-860.
9. Mountifield, R., Bampton, P., Prosser, R., Muller, K. and Andrews, J.M., 2009. Fear and fertility in inflammatory bowel disease: a mismatch of perception and reality affects family planning decisions. Inflammatory bowel diseases, 15(5), pp.720-725.
10. Marri, S.R., Ahn, C. and Buchman, A.L., 2007. Voluntary childlessness is increased in women with inflammatory bowel disease. Inflammatory bowel diseases, 13(5), pp.591-599.
11. Mårdh, P.A., 2004. Tubal factor infertility, with special regard to chlamydial salpingitis. Current opinion in infectious diseases, 17(1), pp.49-52.
12. Tavernier, N., Fumery, M., Peyrin-Biroulet, L., Colombel, J.F. and Gower-Rousseau, C., 2013. Systematic review: fertility in non-surgically treated inflammatory bowel disease. Alimentary pharmacology & therapeutics, 38(8), pp.847-853.
13. Selinger, C.P., Ghorayeb, J. and Madill, A., 2016. What factors might drive voluntary childlessness (VC) in women with IBD? Does IBD-specific pregnancy-related knowledge matter?. Journal of Crohn's and Colitis, 10(10), pp.1151-1158.
14. Gianotten, J., Lombardi, M.P., Zwinderman, A.H., Lilford, R.J. and van der Veen, F., 2004. Idiopathic impaired spermatogenesis: genetic epidemiology is unlikely to provide a short-cut to better understanding. Human reproduction update, 10(6), pp.533-539.
15. Huang, V.W., Chang, H.J., Kroeker, K.I., Goodman, K.J., Hegadoren, K.M., Dieleman, L.A. and Fedorak, R.N., 2015. Does the level of reproductive knowledge specific to inflammatory bowel disease predict childlessness among women with inflammatory bowel disease?. Canadian Journal of Gastroenterology and Hepatology, 29, pp.95-103.
16. Allocca, M., Gilardi, D., Fiorino, G., Furfaro, F., Peyrin-Biroulet, L. and Danese, S., 2018. Sexual and reproductive issues and inflammatory bowel disease: a neglected topic in men. European journal of gastroenterology & hepatology, 30(3), pp.316-322.
17. Lee, H.H., Bae, J.M., Lee, B.I., Lee, K.M., Wie, J.H., Kim, J.S., Cho, Y.S., Jung, S.A., Kim, S.W., Choi, H. and Choi, M.G., 2020. Pregnancy outcomes in women with inflammatory bowel disease: a 10-year nationwide population-based cohort study. Alimentary pharmacology & therapeutics, 51(9), pp.861-869.

18. Ferraro, D., Simone, A.M., Adani, G., Vitetta, F., Mauri, C., Strumia, S., Senesi, C., Curti, E., Baldi, E., Santangelo, M. and Montepietra, S., 2017. Definitive childlessness in women with multiple sclerosis: a multicenter study. Neurological Sciences, 38, pp.1453-1459.

19. Leenhardt, R., Rivière, P., Papazian, P., Nion-Larmurier, I., Girard, G., Laharie, D. and Marteau, P., 2019. Sexual health and fertility for individuals with inflammatory bowel disease. World Journal of Gastroenterology, 25(36), p.5423.

20. Kashkooli, S.B., Andrews, J.M., Roberts, M.B., Selinger, C.P. and Leong, R.W., 2015. Inflammatory bowel disease-specific pregnancy knowledge of gastroenterologists against general practitioners and obstetricians. United European gastroenterology journal, 3(5), pp.462-470.

21. Purewal, S., Chapman, S., Czuber-Dochan, W., Selinger, C., Steed, H. and Brookes, M.J., 2018. Systematic review: the consequences of psychosocial effects of inflammatory bowel disease on patients′ reproductive health. Alimentary Pharmacology & Therapeutics, 48(11-12), pp.1202-1212.

22. Giudice, L.C. and Kao, L.C., 2004. Endometriosis. Lancet, 364, pp.1789-1799.

23. Dunselman, G.A.J., Vermeulen, N., Becker, C., Calhaz-Jorge, C., D'Hooghe, T., De Bie, B., Heikinheimo, O., Horne, A.W., Kiesel, L., Nap, A. and Prentice, A., 2014. ESHRE guideline: management of women with endometriosis. Human reproduction, 29(3), pp.400-412.

24. Norman, R.J., Dewailly, D., Legro, R.S. and Hickey, T.E., 2007. Polycystic ovary syndrome. The Lancet, 370(9588), pp.685-697.

25. Legro, R.S., Arslanian, S.A., Ehrmann, D.A., Hoeger, K.M., Murad, M.H., Pasquali, R. and Welt, C.K., 2013. Diagnosis and treatment of polycystic ovary syndrome: an Endocrine Society clinical practice guideline. The Journal of Clinical Endocrinology & Metabolism, 98(12), pp.4565-4592.

26. Vercellini, P., Viganò, P., Somigliana, E. and Fedele, L., 2014. Endometriosis: pathogenesis and treatment. Nature Reviews Endocrinology, 10(5), pp.261-275.

27. Goodarzi, M.O., Dumesic, D.A., Chazenbalk, G. and Azziz, R., 2011. Polycystic ovary syndrome: etiology, pathogenesis and diagnosis. Nature reviews endocrinology, 7(4), pp.219-231.

28. Green, P.H. and Jabri, B., 2003. Coeliac disease. The Lancet, 362(9381), pp.383-391.

29. Walldorf, J., Brunne, S., Gittinger, F.S. and Michl, P., 2018. Family planning in inflammatory bowel disease: childlessness and disease-related concerns among female patients. European journal of gastroenterology & hepatology, 30(3), pp.310-315.

30. Winter, R.W., Boyd, T., Chan, W.W., Levy, A.N. and Friedman, S., 2022. Risk Factors for Voluntary Childlessness in Men and Women With Inflammatory Bowel Disease. Inflammatory Bowel Diseases, 28(12), pp.1927-1931.

31. Mañosa, M., Navarro-Llavat, M., Marín, L., Zabana, Y., Cabré, E. and Domènech, E., 2013. Fecundity, pregnancy outcomes, and breastfeeding in patients with inflammatory bowel disease: a large cohort survey. Scandinavian journal of gastroenterology, 48(4), pp.427-432.

32. Baird, D.D., Narendranathan, M. and Sandler, R.S., 1990. Increased risk of preterm birth for women with inflammatory bowel disease. Gastroenterology, 99(4), pp.987-994.

33. Ellul, P., Zammit, S.C., Katsanos, K.H., Cesarini, M., Allocca, M., Danese, S., Karatzas, P., Moreno, S.C., Kopylov, U., Fiorino, G. and Torres, J., 2016. Perception of reproductive health in women with inflammatory bowel disease. Journal of Crohn's and Colitis, 10(8), pp.886-891.

34. Vieujean, S., De Vos, M., Paridaens, K., Daftary, G.S., Danese, S. and Peyrin-Biroulet, L., 2022. Fertility and assisted reproductive technologies outcomes of women with non-

surgically managed inflammatory bowel diseases: a systematic review. Journal of Crohn's and Colitis.

35. Ahlfield, J.E., Soler, N.G. and Marcus, S.D., 1985. The young adult with diabetes: impact of the disease on marriage and having children. Diabetes Care, 8(1), pp.52-56.
36. Escott-Price, V., Pardiñas, A.F., Santiago, E., Walters, J., Kirov, G., Owen, M.J. and O'Donovan, M.C., 2019. The relationship between common variant schizophrenia liability and number of offspring in the UK Biobank. American Journal of Psychiatry, 176(8), pp.661-666.
37. Lawn, R.B., Sallis, H.M., Taylor, A.E., Wootton, R.E., Smith, G.D., Davies, N.M., Hemani, G., Fraser, A., Penton-Voak, I.S. and Munafo, M.R., 2019. Schizophrenia risk and reproductive success: a Mendelian randomization study. Royal Society Open Science, 6(3), p.181049.
38. Yates WR, Meller WH, Lund BC, Thurber S, Grambsch PL. Early-onset Major Depressive Disorder in men is associated with childlessness. Journal of affective disorders. 2010 Jul 1;124(1-2):187-90.
39. Goldscheider, F.K. and Kaufman, G., 1996. Fertility and commitment: Bringing men back in. Population and Development Review, 22, pp.87-99.
40. Hellstrand, J., J. Nisén, and M. Myrskylä. 2020. All-time low period fertility in Finland: Demographic drivers, tempo effects, and cohort implications, Population Studies. doi:https://doi.org/10.1080/00324728.2020.1750677.
41. Andersson, L., 2021. Lifetime parenthood in the context of single-and multiple-partner fertility. Advances in Life Course Research, 47, p.100355.
42. Jalovaara, M., L. Andersson and A. Miettinen. (2022). Parity disparity: Educational differences in Nordic fertility across parities and number of reproductive partners, Population Studies, 76(1): 119-136. https://doi.org/10.1080/00324728.2021.1887506.
43. Rotkirch A, Miettinen A. Childlessness in Finland. In: Kreyenfeld M, Konietzka D, eds. Childlessness in Europe: Contexts, causes, and consequences. Cham: Springer, 2017: 139-158.
44. Jalovaara, M., G. Neyer, G. Andersson, J. Dahlberg, L. Dommermuth, P. Fallesen, and T. Lappegård. 2018. Education, gender, and cohort fertility in the Nordic countries, European Journal of Population 35(3): 563–586. doi:https://doi.org/10.1007/s10680-018-9492-2.
45. Holland, J. A., and E. Thomson. 2011. Stepfamily childbearing in Sweden: Quantum and tempo effects, 1950–99, Population Studies 65(1): 115–128. doi:https://doi.org/10.1080/00324728.2010.543693.
46. Wood, J., Neels, K. and Kil, T., 2014. The educational gradient of childlessness and cohort parity progression in 14 low fertility countries. Demographic research, 31, pp.1365-1416.
47. Schoen, R. 2006. Insights from parity status life tables for the 20th century U.S., Social Science Research 35(1): 29–39. doi:https://doi.org/10.1016/j.ssresearch.2004.06.002.
