# Supplementary material for: Evidence from Finland and Sweden on the relationship between early-life diseases and lifetime childlessness in men and women
Source: Nat Hum Behav. 2023 Dec 18;8(2):276–87. doi: 10.1038/s41562-023-01763-x (PMC10896715; doi:10.1038/s41562-023-01763-x)
Supplement: Supplementary file 2 — Reporting Summary [file 41562_2023_1763_MOESM2_ESM.pdf]

nature portfolio

Corresponding author(s): NATHUMBEHAV-22082041C

Last updated by author(s): Oct 4, 2023

Reporting Summary

Nature Portfolio wishes to improve the reproducibility of the work that we publish. This form provides structure for consistency and transparency in reporting. For further information on Nature Portfolio policies, see our Editorial Policies and the Editorial Policy Checklist.

Statistics

For all statistical analyses, confirm that the following items are present in the figure legend, table legend, main text, or Methods section.

n/a	Confirmed	
☐	☒	The exact sample size (*n*) for each experimental group/condition, given as a discrete number and unit of measurement
☐	☒	A statement on whether measurements were taken from distinct samples or whether the same sample was measured repeatedly
☐	☒	The statistical test(s) used AND whether they are one- or two-sided<br>*Only common tests should be described solely by name; describe more complex techniques in the Methods section.*
☐	☒	A description of all covariates tested
☐	☒	A description of any assumptions or corrections, such as tests of normality and adjustment for multiple comparisons
☐	☒	A full description of the statistical parameters including central tendency (e.g. means) or other basic estimates (e.g. regression coefficient) AND variation (e.g. standard deviation) or associated estimates of uncertainty (e.g. confidence intervals)
☐	☒	For null hypothesis testing, the test statistic (e.g. *F*, *t*, *r*) with confidence intervals, effect sizes, degrees of freedom and *P* value noted<br>*Give P values as exact values whenever suitable.*
☒	☐	For Bayesian analysis, information on the choice of priors and Markov chain Monte Carlo settings
☒	☐	For hierarchical and complex designs, identification of the appropriate level for tests and full reporting of outcomes
☒	☐	Estimates of effect sizes (e.g. Cohen's *d*, Pearson's *r*), indicating how they were calculated

Our web collection on statistics for biologists contains articles on many of the points above.

Software and code

Policy information about availability of computer code

Data collection	No code was used to collect data in the study.
Data analysis	For the sibling-based analysis and population-bases analysis, we conducted a conditional logistic regression using the clogit function of the survival_3.2-13 package.<br>For the sensitivity analysis using a time-varying cox PH model, We used the coxph function of the survival_3.2-13 package.<br>When meta-analyzing estimates from two populations, we used the metagen function of the meta_5.1-0 package.<br>For the mediation analysis, we used the CDMA and the VAR.D developed by [PMID: 32608110].<br>For all above statistical analyses, we used R version 4.1.2.

For manuscripts utilizing custom algorithms or software that are central to the research but not yet described in published literature, software must be made available to editors and reviewers. We strongly encourage code deposition in a community repository (e.g. GitHub). See the Nature Portfolio guidelines for submitting code & software for further information.

Data

Policy information about availability of data

All manuscripts must include a data availability statement. This statement should provide the following information, where applicable:

- Accession codes, unique identifiers, or web links for publicly available datasets
- A description of any restrictions on data availability
- For clinical datasets or third party data, please ensure that the statement adheres to our policy

Data availability statement:
Due to data protection regulations, we are not allowed to share individual-level data directly. However, all Finnish and Swedish register data used in this study can be applied from national data agencies. All Finnish and Swedish register data used in this study can be applied from national data agencies including Statistics Finland (https://www.stat.fi/index_en.html), Population Information System (DVV, https://dvv.fi/en/individuals), Finnish Institute for Health and Welfare (THL, https://thl.fi/en/web/thlfi-en/statistics-and-data/data-and-services/register-descriptions/care-register-for-health-care) and Finnish Cancer Registry (https://cancerregistry.fi/) from Finland, and Statistics Sweden (https://www.scb.se/en/) and National Board of Health and Welfare (Socialstyrelsen, https://www.socialstyrelsen.se/en/) from Sweden.
All results (aggregated data) can be explored on the interactive online dashboard available at https://dsgelrs.shinyapps.io/DiseaseSpecificLRS/.

Human research participants

Policy information about studies involving human research participants and Sex and Gender in Research.

Reporting on sex and gender	We collected the information of sex for each participant from the nationwide population system. The analysis has been done separately for two sexes. Given that the previous studies mainly focus on women and lack of evidence for men, we further investigated the sex-difference for each identified association.
Population characteristics	We examined all individuals born in Finland (n=1,035,928) and Sweden (n=1,509,092) between 1956 and 1968 (men) or 1956 and 1973 (women) and followed them up until the end of 2018, when most have completed their reproductive lifespan. Socio-demographic, health, and reproductive information was obtained from nationwide registers.<br>For the entire study population, we have information from nationwide registers covering 414 disease diagnoses across 16 main categories.
Recruitment	We considered all individuals born in Finland (n=1,035,928) and Sweden (n=1,509,092) between 1956 and 1968 (men) or 1956 and 1973 (women) and followed them up until the end of 2018. Therefore, no selection has been done in terms of participant recruitment.
Ethics oversight	Ethics committee/IRB of Regional Ethical Review Board in Uppsala gave ethical approval for this work (2018/223). The use of Swedish registry data for this study is approved by socialstyrelsen (permit number: 27035/2018) and Statistics Sweden (permit number: 247849). The use of Finnish registry data is approved by Digital and population data service agency (permit numbers: VRK/6551/2019-1 and VRK/6551/2019-2), Statistics Finland (permit number: TK-53-1813-19), and Finnish Institute for Health and Welfare (permit number: THL/804/5.05.00/2019).

Note that full information on the approval of the study protocol must also be provided in the manuscript.

Field-specific reporting

Please select the one below that is the best fit for your research. If you are not sure, read the appropriate sections before making your selection.

☒ Life sciences ☐ Behavioural & social sciences ☐ Ecological, evolutionary & environmental sciences

For a reference copy of the document with all sections, see nature.com/documents/nr-reporting-summary-flat.pdf

Life sciences study design

All studies must disclose on these points even when the disclosure is negative.

Sample size	We defined our main outcome, lifetime childlessness, as individuals that have had no live-born children by the end of their reproductive lifespan (age 45 for women; 50 for men). To have virtually complete coverage of health and reproductive information until the end of reproductive period, we examined all individuals born 1956-1968 (men) and 1956-1973 (women) in Finland (n=1,035,928) and Sweden (n=1,509,092) to completion of their reproductive lifespan in 2018 (age 45 for women and 50 for men). For these index individuals, we also obtained information for parents, spouses, siblings, and children for a total of 9,305,692 individuals (3,640,464 Finns and 5,665,228 Swedes). With the unique datasets, we were able to analyze 414 diseases for which we had more than 30 affected individuals in the sibling-based analysis for each sex, in Finland or Sweden.
Data exclusions	Individuals who emigrated during the study period were excluded to avoid incomplete follow-up for disease diagnoses and reproductive information. We further excluded individuals who died before the age of 16 to eliminate the impact of diseases on pre-reproductive survival.

	We excluded children conceived by assisted reproductive techniques (0.3% Finland, 0.8% Sweden) to control for potential confounding from social inequalities in medical help-seeking for infertility, especially during the observational period.
Replication	The use of nationwide data from two countries provided a large sample of 2.5 million and allowed us to assess how robust our findings were to different healthcare systems and diagnostic practices. The identified associations with diseases such as type 1 diabetes and several major mental health disorders were consistent with the previous studies [PMID: 17563340; 23147713].
Randomization	No randomization was performed because we considered everyone from Finland and Sweden born in certain years.
Blinding	Blinding was not relevant to the study because this was a population-based study, with all analyzed data obtained from nationwide registers.

Reporting for specific materials, systems and methods

We require information from authors about some types of materials, experimental systems and methods used in many studies. Here, indicate whether each material, system or method listed is relevant to your study. If you are not sure if a list item applies to your research, read the appropriate section before selecting a response.

Materials & experimental systems

n/a	Involved in the study
☒	☐ Antibodies
☒	☐ Eukaryotic cell lines
☒	☐ Palaeontology and archaeology
☒	☐ Animals and other organisms
☒	☐ Clinical data
☒	☐ Dual use research of concern

Methods

n/a	Involved in the study
☒	☐ ChIP-seq
☒	☐ Flow cytometry
☒	☐ MRI-based neuroimaging
